# Strategies for improving the genome-editing efficiency of class 2 CRISPR/Cas system

**DOI:** 10.1016/j.heliyon.2024.e38588

**Published:** 2024-09-27

**Authors:** Linli Wang, Hongbing Han

**Affiliations:** a Frontiers Science Center for Molecular Design Breeding (MOE), China Agricultural University, Beijing, 100193, China; b Beijing Key Laboratory of Animal Genetic Improvement, College of Animal Science and Technology, China Agricultural University, Beijing, 100193, China; c Key Laboratory of Animal Genetics, Breeding and Reproduction of the Ministry of Agriculture and Rural Affairs, College of Animal Science and Technology, China Agricultural University, Beijing, 100193, China

**Keywords:** CRISPR/Cas, Genome-editing efficiency, gRNA engineering, Exonuclease, Chemical modification

## Abstract

Since its advent, gene-editing technology has been widely used in microorganisms, animals, plants, and other species. This technology shows remarkable application prospects, giving rise to a new biotechnological industry. In particular, third-generation gene editing technology, represented by the CRISPR/Cas9 system, has become the mainstream gene editing technology owing to its advantages of high efficiency, simple operation, and low cost. These systems can be widely used because they have been modified and optimized, leading to notable improvements in the efficiency of gene editing. This review introduces the characteristics of popular CRISPR/Cas systems and optimization methods aimed at improving the editing efficiency of class 2 CRISPR/Cas systems, providing a reference for the development of superior gene editing systems. Additionally, the review discusses the development and optimization of base editors, primer editors, gene activation and repression tools, as well as the advancement and refinement of compact systems such as IscB, TnpB, Fanzor, and Cas12f.

## 1 Introduction

Gene editing refers to biological genome modifications that result in the deletion, insertion, or replacement of targeted DNA sequences. In recent years, this technology has made outstanding achievements in basic research in the fields of life sciences, clinical treatments, and diagnostics. The clustered regularly interspaced short palindromic repeats-CRISPR-associated protein (CRISPR/Cas) system, which consists of RNA and Cas proteins, is an adaptive immune system based on RNA-mediated nuclease cleavage to remove foreign nucleic acids in microorganisms [1,2]. The main principle of gene editing involves creating double-stranded breaks (DSBs) in the genome induced by the CRISPR/Cas system, followed by homologous directed repair using exogenous donor DNA or nonhomologous end joining (NHEJ) to knock out the target gene via random insertion-deletion mutation. The CRISPR/Cas system has been widely used in the treatment of diseases such as cancer [3,4], liver diseases [[5], [6], [7]], and cardiovascular diseases [8,9]. In December 2023, the US Food and Drug Administration (FDA) approved a therapy for sickle cell disease in patients aged 12 years and older, known as Casgevy, which is the first CRISPR-based gene therapy. The FDA approved clinical trials of CRISPR-based therapies for HIV infections (EBT-101) in September 2021 and Duchenne muscular dystrophy (CRD-TMH-001) in August 2022. A CRISPR-based severe acute respiratory syndrome coronavirus 2 (SARS-CoV-2) test kit was approved by the FDA for the emergency diagnosis of coronavirus disease in 2021 [10,11]. In biological breeding, a series of varieties have been created using gene-editing technology. Pigs lacking functional CD163 were generated using CRISPR/Cas technology, inducing resistance to porcine reproductive and respiratory syndrome virus infections [12]. Researchers have inserted natural resistance-associated macrophage protein 1 into the genome of bovine fetal fibroblasts using Cas9 nickase to obtain cattle with high resistance to *Mycoplasma bovis* infections [13]. Myostatin is a negative regulator of skeletal muscle growth; a series of myostatin-knockout animals, such as pigs, sheep, and rabbits, were produced with the “double muscle” phenotype using the CRISPR/Cas9 system [[14], [15], [16], [17]].

Currently, the CRISPR/Cas system is divided into two main classes: class 1 and class 2. CRISPR/Cas9, belonging to class 2, is the most widely used CRISPR/Cas system and has excellent gene editing efficiency. However, the editing efficiency of CRISPR/Cas9 can be improved further [18,19]. Other CRISPR/Cas systems, such as Cas12a (Cpf1), Cas12i, Cas12f, Cas12e (CasX), Cas12b, Cas12a2, Cas13a, and Cascade-Cas3, are functionally distinct from Cas9 effectors [[20], [21], [22], [23], [24], [25], [26], [27], [28], [29], [30], [31], [32], [33]]. Compared with the classical nuclease *Streptococcus pyogenes* Cas9 (SpCas9) with 1368 amino acids, the other CRISPR effectors with smaller sizes, such as Cas12i (1033–1093 amino acids), CasMINI (529 amino acids), and IscB (450 amino acids), are more suitable for viral vector delivery [20,22,30,31]. Cas9 requires two small RNAs, CRISPR RNA (crRNA) and transactivating crRNA (tracrRNA), whereas other CRISPR effectors, such as Cas12a, Cas12i, and Cas12j, require a single crRNA as a guide RNA (gRNA) [20,27,31,34,35]. Additionally, Cas9 induces DSBs in the region upstream and proximal to protospacer-adjacent motif (PAM) sequences; however, other effectors, such as Cas12a, cleave the PAM-distal region. Cas12i and Cas12a recognize 5′-TTN PAM and 5′-TTTV PAM in the target DNA, respectively [20,36]. However, SpCas9 recognizes 5′-NGG PAM. Some effectors, such as Cas12a and Cas12j, are suitable for multigene editing by autonomously processing precursor crRNA (pre-crRNA) to form mature crRNA, owing to their RNase activity. Cas12i can accommodate crRNA-target DNA heteroduplex lengths of 28 bp compared to 20 bp for SpCas9, which may indicate that Cas12i has a lower off-target effect [20]. Some new systems can target RNA and single-stranded DNA (ssDNA) cleavage [25,28]. Despite these advantages, the new systems are not used as widely as expected, partly because of the overall low editing efficiency. Overcoming this disadvantage could lead these new CRISPR/Cas systems to become widely used tools in experimental and clinical applications.

**Fig. 1:**
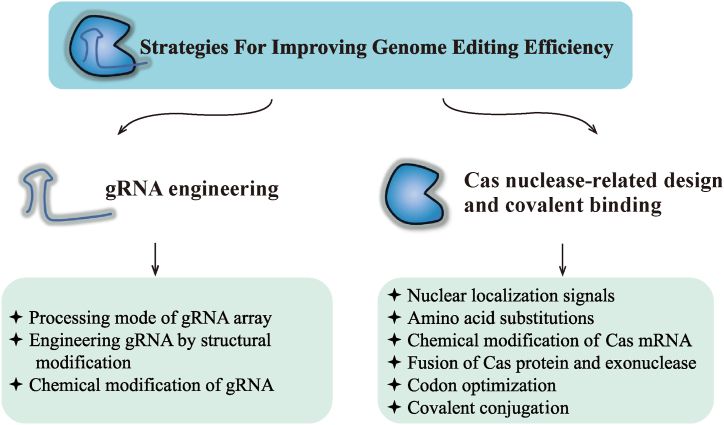
Effective methods to improve editing efficiency using gRNA engineering and Cas nuclease-related design and covalent binding. gRNA engineering involves three aspects: gRNA array processing, gRNA structural engineering, and gRNA chemical modification. Cas nuclease-related design and covalent binding include nuclear localization signals, amino acid substitutions, chemical modification of Cas mRNA, fusion expression of Cas protein and exonuclease, codon optimization, and covalent conjugation. gRNA:guide RNA; Cas: CRISPR-associated proteins.

This review introduces the representative CRISPR/Cas editing systems. Moreover, it focuses on methods for achieving higher editing efficiency by improving gRNA and Cas nuclease to provide a reference for the optimization of an excellent gene-editing system (Fig. 1).

## 2 CRISPR/Cas system

**Fig. 2:**
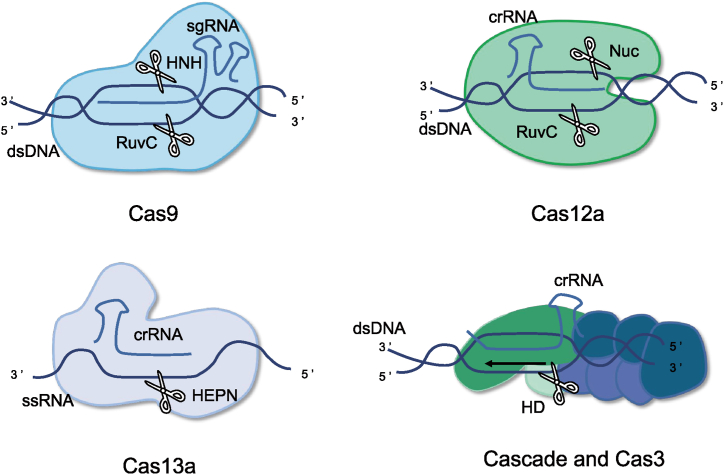
Overview of representative CRISPR/Cas gene editing system. Cas9 and Cas12a are used to cut dsDNA; Cas9 produces a blunt end whereas Cas12a produces a staggered end. Cas13a recognize and cleave ssRNA. Cascade and Cas3 mainly produce large deletions of dsDNA in the presence of recognizable dsDNA substrates. Cas: CRISPR-associated proteins; dsDNA: double-stranded DNA; sgRNA: single guide RNA; ssRNA: single-stranded RNA; crRNA: CRISPR RNA; HEPN: higher eukaryotic and prokaryotic nucleotide-binding; HD: histidine-aspartate.

The CRISPR/Cas system, an adaptive immune system in prokaryotes against foreign exogenous genetic elements, such as phages or plasmids, is mainly composed of crRNA, tracrRNA, and Cas proteins. The crRNA-tracrRNA-Cas protein complex recognizes the appropriate PAM in a DNA target. The gRNA sequence matches the complementary DNA sequence, guides the nuclease to the correct position, and determines the gene editing specificity. Cas protein residues are responsible for target strand cleavage. The CRISPR/Cas system is now divided into class 1 and class 2, including a total of six types (I, III, and IV belonging to class 1, and II, V, and VI belonging to class 2) [37]. The effector of class 1 for cutting nucleic acids is a complex formed by multiple Cas proteins, such as the type I-C [38,39] and type I-E systems [40,41], whereas the effector of class 2 is a single Cas protein, such as the CRISPR/Cas9 and CRISPR/Cas12a systems. Type II CRISPR/Cas9 and Type V CRISPR/Cas12a systems are the most widely used for genome editing because of their high efficiency and convenience. Representative CRISPR/Cas systems are shown in Fig. 2 and Table 1.

**Table 1:** Characteristics of representative CRISPR/Cas systems.

Effector	Type	Nuclease domain	gRNA	Substrate	PAM/PFS	Indel type	Cleavage pattern	Reference
Cas9	II	HNH and RuvC	crRNA and tracrRNA	dsDNA	3′ GC-rich PAM	small indels	blunt end	[42,43]
Cas12a	V	RuvC and Nuc (putative)	crRNA	dsDNA	5′ AT-rich PAM	small indels	staggered end	[44,45]
Cas13a	VI	HEPN	crRNA	RNA	3′ PFS: non-G	degraded RNA	depends on local target sequence	[46,47]
Cascade and Cas3	I	HD	crRNA	dsDNA	variable	large fragment deletion	degrades NTS DNA	[39,41,48]

Cas: CRISPR-associated proteins; CRISPR: clustered regularly interspaced short palindromic repeats; crRNA: CRISPR RNA; dsDNA: double-stranded DNA; gRNA: guide RNA; HEPN: higher eukaryotic and prokaryotic nucleotide-binding; indel: insertion or deletion; NTS: non-targeted strand; PAM: protospacer-adjacent motif; PFS: protospacer flanking sites; ssDNA: single-stranded DNA; ssRNA: single-stranded RNA; tracrRNA: transactivating crRNA; HD: Histidine-Aspartate.

### 2.1 CRISPR/Cas9

The CRISPR/Cas9 system is the most widely used and is composed of crRNA, tracrRNA, and Cas9 nucleases. The crRNA and tracrRNA can be fused to form a single guide RNA (sgRNA) [49]. Cas9 nuclease is composed of a recognition (REC) lobe and a nuclease (NUC) lobe. The NUC lobe is composed of RuvC and HNH domains, which are responsible for cutting double strands of DNA to produce blunt ends. Some Cas9 nucleases can also produce 1–2 bp staggered ends. SpCas9, the most commonly used Cas9, recognizes a short 5′-NGG PAM. The preferred PAM sequence of SpCas9 is found on average every 8 bp in the genome. A Cas9 variant with more targets has been designed, and a near-PAMless Cas9 variant, named SpRY, has been derived, which can effectively cut arbitrary DNA sequences in vitro [50,51]. In addition, other Cas9 nucleases have distinct advantages, such as smaller size and PAMs rich in pyrimidine (Nme2Cas9) [52]. The CRISPR/Cas9 system is highly efficient and widely used in animal and plant breeding programs, gene therapy, and medical research, and has greatly promoted scientific progress.

### 2.2 CRISPR/Cas12a

The CRISPR/Cas12a system (previously known as CRISPR/Cpf1) is widely used in addition to CRISPR/Cas9 [36]. Cas12a homologs from *Acidaminococcus* (AsCas12a) and *Lachnospiraceae* (LbCas12a) have been demonstrated to be active in mammalian cells. The CRISPR/Cas12a system is composed of a crRNA and a Cas12a nuclease. With its RNase III function, Cas12a can process pre-crRNA into crRNA, independently targeting multiple loci and facilitating multiplex genome editing. TTTV (where V is A, C, or G) is a typical PAM used by CRISPR/Cas12a. The 23rd nucleotide downstream of PAM and the 18th nucleotide of the non-target strand are cleaved by the Nuc and RuvC domains of Cas12a, respectively, to form the 5 nt staggered end, which is in sharp contrast to the blunt end produced by Cas9. The incision of the CRISPR/Cas12a system is far from the PAM site and can be recognized and cut multiple times; therefore, multiple gene edits can be performed at the same location, which may improve the efficiency of gene knock-in Ref. [53]. Additionally, the CRISPR/Cas12a system has low off-target rates, which is conducive for gene editing with high efficiency and safety [32,33].

### 2.3 CRISPR/Cas13a

The CRISPR/Cas13a system (previously known as CRISPR/C2c2) is a gene-editing system that specifically binds and cleaves RNA. Cas13a requires a 24-base crRNA that interacts with Cas13a via a uracil-rich stem-loop structure and facilitates target cleavage through conformational changes. Cas13a recognizes the protospacer flanking sites (PFS) located at the 3′ end of the spacer region, consisting of a single A, U, or C. The Cas13a nuclease contains two conserved higher eukaryotic and prokaryotic nucleotide-binding (HEPN) domains. Once the Cas13a-crRNA complex recognizes the PFS, the crRNA binds to complementary regions in the target RNA, inducing a co-conformational change in Cas13a. The Cas13a-crRNA complex in the non-enzymatic state transforms to exhibit HEPN-RNase activity, and the target RNA is cis-cleaved to achieve RNA knockout. The nearby single-stranded RNA is trans-cleaved in a non-specific manner, regardless of whether it is homologous to crRNA or whether there is a PFS. Based on the CRISPR/Cas13a system, nucleic acid detection, termed specific high-sensitivity enzymatic reporter unlocking (SHERLOCK) and developed by Kellner et al., is faster and less device-dependent than traditional qPCR [54]. Additionally, dCas13a was obtained from the Cas13a protein with a mutation in the R-XXXX-H motif of the HEPN domain. dCas13a lacks cleavage enzyme activity but retains the binding ability. The ability to specifically recognize and bind to target RNA without cutting enables dCas13 to be applied to RNA manipulation, such as RNA imaging and transcriptome regulation, through fusion with a variety of functional proteins [[55], [56], [57]].

### 2.4 Cascade-Cas3

The CRISPR/Cas3 systems are the most common CRISPR systems in nature. Cas3, with helicase and nuclease domains, in cooperation with the cascade complex, cleaves target DNA. This cascade recognizes dsDNA, leading to the formation of an RNA-DNA-hybrid-containing R-loop. Cas3 is then recruited to the cascade/R-loop to degrade the target DNA. These CRISPR/Cas systems, which produce large and unfixed DNA deletions, can be used to target and degrade foreign genetic elements from bacteria, or to study gene function and chromosome variation. In addition, they can be used for the rapid detection of nucleic acids and to combat antimicrobial resistance [[58], [59], [60]].

## 3 gRNA engineering

**Fig. 3:**
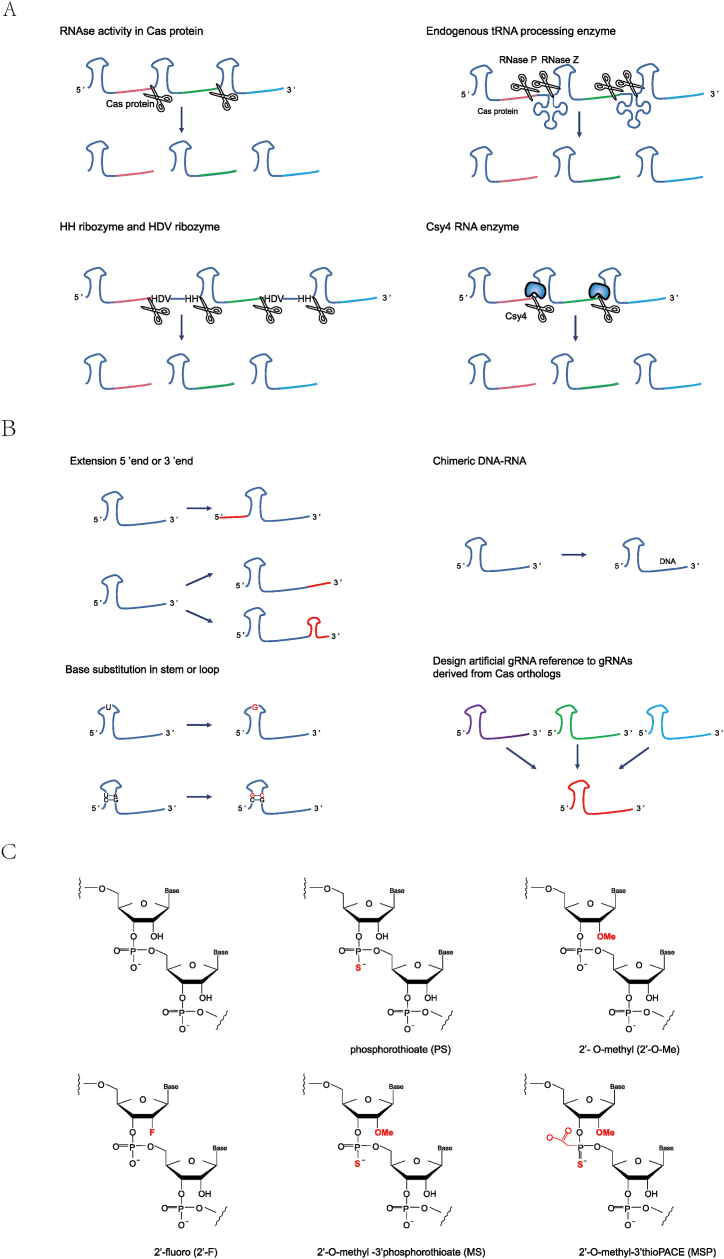
Representative processes related to gRNA to improve editing efficiency. (A) crRNA array processing. For multiple targets, the crRNA array can generally be processed by the RNA enzyme activity of Cas (for example, most type V Cas proteins), endogenous tRNA processing enzymes, ribozymes (commonly including HH and HDV ribozymes), and Csy4 RNA enzyme. (B) gRNA engineering by structural modification. Common modifications include extending the 5′ or 3′ terminal, base substitution in the stem-loop structure, formation of chimeric DNA-RNA by replacing part of RNA with DNA, and re-designing artificial gRNA reference to gRNAs derived from Cas orthologs. (C) Chemical modification of crRNA. This mainly involves the modification of the phosphoric acid backbone, namely, PS modification, and sugar modification, mainly including 2′-O-Me, 2′-F, MS, and MSP modifications. Cas: CRISPR-associated proteins; tRNA: transfer RNA; HH: hammerhead; HDV: hepatitis deltavirus; gRNA: guide RNA; PS: phosphorothioate; 2′-O-Me: 2′-O-methyl; 2′-F: 2′-fluoro; MS: 2′-O -methyl -3′phosphorothioate; MSP: 2′-O-methy1-3′thioPACE.

Two components of gene editing in the CRISPR/Cas system, gRNA and Cas proteins, have been used to improve genome-editing efficiency. This review, for the first time, summarizes the methods commonly used for optimizing gRNA to improve editing efficiency; this optimization mainly involves three aspects: gRNA array processing, gRNA structural engineering, and chemical modification of gRNA (Fig. 3).

### 3.1 Processing mode of gRNA array

(1) HH and HDV ribozymes

The expression of crRNAs with non-purine beginnings is less efficient because, initially, RNA polymerase III (Pol III) promoters, which drive crRNA expression, favor pyrimidines (G/A). Additionally, at the 3′ terminals, Pol III terminates at six consecutive Ts forming a termination signal, generating a crRNA with variable U-tails of 1–6 nucleotides. However, such U-tails can have a significant negative effect on the crRNA activity of AsCpf1 and reduce editing efficiency [61]. Therefore, the precise expression of crRNA is a key determinant in maintaining CRISPR/Cas activity. Two ribozymes, hammerhead (HH) and hepatitis deltavirus (HDV) ribozyme, have been used to assemble the RGR (ribozyme-gRNA-ribozyme) system. In this system, these two ribozymes are placed at the 5′ and 3′ ends of gRNA, catalyzing the cleavage of the RNA substrate at a specific phosphodiester bond and yielding the mature gRNA from gRNA precursors [62]. HH and HDV ribozymes can process crRNA transcripts to produce precise crRNA complexes with improved editing efficiency. The self-cleavage activities of ribozymes enable coordinated expression of crRNA without additional poly U sequences at the 3′ terminal, significantly improving the efficiency of gene editing [63,64]. The self-cleaving activity of HDV ribozymes can be modified by designing double-pseudoknot HDV ribozymes using an inverse RNA-folding algorithm [65]. This method can be used to improve the efficiency of multigene editing using the CRISPR/Cas system. Multiple crRNAs can be processed by ribozymes. However, the decrease in processing activity as the number of gRNAs increases is a potential disadvantage of ribozyme-mediated gene editing [66].

(2) Endogenous tRNA processing

Transfer RNA (tRNA) is a small RNA molecule that is widely present in living organisms and carries and transports amino acids. tRNAs are encoded by short strands of 70–90 nucleotides in length and are folded into a clover shape. The precursors of tRNA are cleaved at specific eukaryotic sites by RNase P and RNase Z (or RNase E in bacteria), only requiring the acceptor stem of tRNA, the D loop arm, and the TψC loop arm. tRNA is one of the most abundant cellular components; thus, processing systems should be capable of handling numerous substrates. In addition, the tRNA gene contains internal promoter elements (boxes A and B) that recruit the Pol III complex and may act as a potential transcriptional enhancer of Pol III. Based on these characteristics, endogenous tRNA systems can be designed as universal platforms for accurate gRNA processing. Two or more gRNA sequences can be serially connected to the same expression vector via tRNA. The endogenous tRNA processing system can be used to generate numerous gRNAs carrying target sequences, which guide the Cas nuclease to edit multiple sites and improve mutation efficiency. Endogenous tRNA systems can improve genome-editing capacity and efficiency without introducing nucleases or RNA beyond Cas9/gRNA. This system has been designed as a simple and powerful platform to improve the targeting and multiple editing capabilities of the CRISPR/Cas9 system [67] and is widely used in plants [66], yeast, mammalian cells [68,69], and zebrafish [70]. Human cysteine tRNA (hCtRNA), 75 nt in length, has been screened for single-base editing at up to 31 sites and prime editing at up to three sites [71]. RNase P and RNase Z cleave tRNA precursors at the 5′ and 3′ ends, respectively. RNase P, composed of RNA and protein subunits, varies in complexity based on biological functions. The RNA subunit is the catalytic center, and the protein subunit is a key cofactor [72]. The efficiency of cleavage may be affected by these two factors. Furthermore, RNase Z is a solely protein-based enzyme whose efficiency is determined by the structure of the substrate. If the 3′ end of the tRNA precursor is not conducive to RNase Z recognition and cleavage, its efficiency may be compromised.

(3) Csy4 RNA enzyme

Csy4 is a bacteria-derived RNA-cutting enzyme. In the bacterial immune system, when the CRISPR gene cluster is transcribed into a precursor, Csy4 processes the precursor into a single mature crRNA through sequence and structure specificity. Csy4 specifically recognizes the 28 nt sequence of the conserved stem-loop and then cleaves the 3′ end of the stem base [73]. Csy4 is a tool for building complex multi-gRNAs without interfering with the endogenous RNA mechanisms of the host cells. In the EGFP destruction assay conducted using human cells, single gRNAs treated with Csy4 were active at ∼90 % of the activity observed for matched gRNAs prepared with standard U6 promoter vectors; therefore, the presence of a Csy4 recognition site at the 3′ end of the gRNA does not appear to impair the CRISPR/Cas9 editing activity [74]. The gene network design of CRISPR/Cas9-based human cells was achieved using the RNA triple-helix structure and Csy4 [75]. FAA1, FAA4, POX1, and TES1 quadruple knockouts were generated using type III CRISPR/Cas-related Csy4 endonuclease from *Pseudomonas aeruginosa* in 24 colonies of yeast. The knockout efficiency was 96 %. In addition, this study effectively regulated three genes (*OLE1*, *HMG1*, and *AGS1*) by applying Csy4 to the CRISPR interference (CRISPRi) [76]. Additionally, Csy4 can be used to construct gRNA arrays with up to 10 gRNAs driven by PoII promoters, and its editing efficiency is significantly higher than that of a single U6-gRNA. Arrays with five gRNAs also showed comparable gene activation [77]. In prime editing, editing efficiency is improved by adding Csy4 recognition sites in the middle of the prime editing guide RNA (pegRNA) to prevent cyclization of the pegRNA itself [78]. In addition, the tRNA length is typically 70–90 nt, and the HH ribozyme is 37 nt, the HDV ribozyme is 68 nt, while the Csy4 recognition site is compact with 28 nt [79]. The concise recognition site is critical for vector capacity, particularly in the case of viral vectors or multiple gRNAs. However, Csy4 requires the construction of additional sequences to express proteins, which may not be convenient and may cause additional unknown effects in cells. The target specificity of gRNA systems processed by Csy4 necessitates system reconstruction when the target changes. The requirement for specific auxiliary proteins in Csy4 function remains unclear and warrants further investigation.

### 3.2 Engineering gRNA by structural modification

Fusing crRNA and tracrRNA into an sgRNA represents a classic example of gRNA engineering, significantly enhancing the practicality and efficiency of the CRISPR-Cas9 genome-editing system [49]. Various strategies for engineering gRNA have been developed. These approaches typically include changes in gRNA sequence length, base insertions or substitutions, addition of structured RNA, and addition of MS2 RNA.

Extension of the 5′ terminal of crRNA improves the editing efficiency of CRISPR/Cpf1. For example, the extension of 59 nucleotides at the 5′ terminal of crRNA significantly improves the efficiency of gene editing in vitro and in vivo [80]. In addition, efficient genome editing has been reported in the CRISPR/Cas12a system using 3′-prominent crRNA. The addition of 8-meric uridinylates (U8) to the 3′ terminals of crRNA in the CRISPR/Cpf1 system increased the indel efficiency from ∼20 % to ∼50 % for synthesized crRNAs in HEK293T cells. For transcribed crRNAs, T4AT6 produced the greatest indel efficiency, which increased from ∼30 % to ∼50 % in HEK293T cells [81]. Extending the length of gRNA enhances editing efficiency, possibly because longer gRNAs take more time to be degraded. Interestingly, shortening the length of gRNA can improve its expression and may facilitate more efficient delivery. Shortening the gRNA of AsCas12f by deletion of stems 3, 4, and 5 revealed a 4.5-fold increase in gRNA expression in HEK293T cells compared with the deletion of stem 5 alone [82]. The enhancement of editing efficiency cannot be determined solely by the length of the gRNA because also depends on the specific context and scenario.

In addition to changes in gRNA length, specific base insertions or substitutions can improve editing efficiency. Replacing the loop in the AsCas12a crRNA with the loop base of Cas12a family orthologs yielded crRNAs with higher activity. When the AsCas12a loop (UCUU) was replaced by its ortholog, Lb2Cas12a loop (UAUU), the efficiency of gene editing significantly improved [83]. Furthermore, in the CRISPR/Cas12a system, three A:U base pairs are found in the stem-loop region of the crRNA direct repeat (DR) sequence, and base-pair mismatch within the stem-loop can directly eliminate Cas12a nuclease activity. In contrast, if the stem-loop base pairing is preserved, such as from A:U pairing to G:C pairing, Cas12a nuclease activity is preserved [36]. Therefore, changing the A:U to G:C pairing can enhance the thermal stability of the crRNA stem-loop, increase the number of crRNAs folded correctly, and improve the efficiency of gene editing without affecting the activity of Cas12a. In addition, by replacing the 3′ end of crRNA with a 12 base DNA to create a chimeric DNA-RNA guide, using this enhanced Cas12a system considerably increased the average induction efficiency of target sequence mutations compared to the WT Cas12a system (1.7–16.9 × ). Further, the target specificity showed an average 2.8-fold increase because the mean mutation induction efficiency of off-target nucleotide sequences was reduced from 0.5–10.6 % to 0.1–3.6 % [84]. Insertions or substitutions of bases in sgRNA may alter its secondary structure or dynamic properties, making it more conducive to interacting with Cas proteins and thereby enhancing overall editing efficiency.

To avoid competition between misfolded and active gRNAs for binding to the Cas9 enzyme, the addition of a locking hairpin with a melting temperature of 71 °C with one of the stems in the tracrRNA increased the cleavage efficiency to 169 % [85]. Furthermore, the stability of SpCas9 sgRNA can be improved by adding a hairpin structure at the 3′ end. Similarly, adding a DR to the 3′ end of AsCas12a crRNA increased the knockout efficiency two-fold [86]. Adding a hairpin structure to the gRNA could improve editing efficiency probably because it increases the amount of correctly folded gRNA or prevents gRNA degradation.

In addition, enhancing editing efficiency can also be achieved through the MS2 and MS2-coat protein (MS2-MCP) system, primarily applied in prime editing, gene activation, and gene knock-in. In prime editing, researchers have significantly boosted the efficiency of prime editor (PE) 3 and PE5 systems by modifying sgRNA with MS2 hairpin structures to recruit MCP-fused P65. The increased efficiency may be due to altered chromatin accessibility [87]. In gene activation, sgRNA hairpin structures were modified to incorporate two MS2 aptamers, each containing two binding sites for the MS2 protein. The MS2-p65-HSF1 fusion protein can be recruited to the MS2 aptamers, theoretically recruiting up to four copies of p65-HSF1, thereby achieving robust transcriptional activation [88]. Regarding gene knock-in, a transcription-coupled Cas9-mediated editing (TEd) approach has been developed, where efficiency may be restricted by the distance between transcription-coupled HR donor DNAs (TC donors) and DSBs. By appending four MS2 aptamers to the 3′ end of TC RNA and fusing MCP with Cas9, gene insertion efficiency was significantly enhanced [89]. The MS2-MCP system facilitates the recruitment of crucial molecules near specific sites, thereby increasing the local concentration of crucial molecules around the target site and enhancing editing efficiency.

All these methods can increase the efficiency of gene editing or provide other advantages while maintaining a comparable editing efficiency; however, choosing which method to apply depends on the situation. The general principles for gRNA design can be summarized as follows: (i) enhancing the internal stability of gRNA to minimize unintended secondary structures, such as replacing A:U pairing in the stem-loop region with more stable G:C pairing, and incorporating high-melting-point locked hairpins; (ii) increasing gRNA abundance, for example, shortening the gRNA sequence to obtain higher expression; (iii) reducing gRNA degradation, such as by adding a DR at the 3′ end of gRNA; and (iv) altering the hybridization energy between gRNA and target DNA, such as by substituting the crRNA region responsible for DNA binding with DNA.

### 3.3 Chemical modification of gRNA

The chemical modification of nucleotides has been a routine practice followed by laboratories and commercial suppliers for several years. The chemical modification of gRNA can occur in the spacer region or repeat sequence. Chemically modified crRNAs can perform superior editing functions in primary cells, stem cells, and other challenging cell lines because chemical modification provides greater stability and protection from exonucleases. Common modifications include 2′-O-methyl (2′-O-Me), 2′-fluoro (2′-F), phosphorothioate (PS), 2′-O-methyl 3′ thioPACE (MSP), 2′-O-methyl-PACE (MP), and other ribose modifications, which reduce the sensitivity to nuclease and immune stimulation and are involved in cell stability.

The 2′-O-Me nucleoside is a naturally occurring RNA analog, and incorporation of the 2′-O-Me group into oligonucleotides increases binding affinity and nuclease resistance. The ribosyl-2′-O-Me U-rich crRNA can improve the digestibility of dsDNA and enable safe and specific genome editing in fertilized mouse eggs by introducing methoxy modification into the ribose of the 3′ protruding end of U-rich crRNA and mixing it with Cas12a protein [90]. Performing 2′-O-Me chemical modification of the Cas9 gRNA in addition to the nexus loop position, increased the editing efficiency from 62 % to 75 % [85].

The 2′-F RNA, which has fluoro at the 2′-ribose site, replaces the natural 2′-OH group. This highly electronegative group is similar in size to the hydrogen atom and drives the conformation toward the C3′-endo sugar pucker, resulting in increased binding affinity [91]. In the CRISPR/Cpf1 system, 2′-F modification was introduced into the five bases of the crRNA 3′ end, which improved the editing efficiency by 127 % compared to the WT crRNA [83].

In addition, chemical modification of the phosphoric acid backbone using PS can significantly increase the resistance of nucleases and improve their function [92]. PS modifications are frequently combined with other modifications to enhance the editing efficiency of CRISPR/Cas systems. Introducing 2′-OMe, 2′-F, and PS modifications into gRNA to guide SpCas9 is more effective than the unmodified counterpart [93]. Similarly, in the CRISPR/Cas9 system, a chemically modified 29-nucleotide synthetic CRISPR RNA (scrRNA) has been developed via modification of PS, 2′-O-Me, and 2′-F, and truncation of a 42-nucleotide crRNA, producing high cleavage activity and preventing degradation by nucleases [94]. Additionally, different crRNAs were obtained using different chemical modifications of three uridine nucleotides at the 3′ end of the 23 nt spacer sequence or by covering the 3′ end with inverted thymidine. These three modifications were 2′-O-methylation (M), phosphorothioate linkage (S), and 2′-O-methylation and 3′ phosphorothioate linkage (MS). All chemical modifications enhanced the knockdown of CRISPR/Cas13, with the M modification having the largest overall knockdown (80 %) [95].

In addition to the commonly used modifications mentioned above for CRISPR/Cas systems, MSP has also been demonstrated to enhance the editing efficiency of CRISPR/Cas systems. In K562 cells, the efficiency of genome editing mediated by the CRISPR/Cas9 system can be significantly improved by modifying the three bases at the 5′ and 3′ ends of crRNA with MS or MSP. K562 cells were cotransfected with 1 μg sgRNA targeting the IL2RG locus and plasmid encoding Cas9. The indel efficiency without modification was 2.4 %, and the efficiencies of MS and MSP were 68.0 % and 75.7 %, respectively. In addition, the efficiency further increased to 75.3 % and 83.3 % after a 20-fold increase in sgRNA [96].

In general, the following guidelines for chemically modifying gRNAs can be determined: (i) the seed region of the gRNA can only be modified in small amounts owing to the poor tolerance to chemical modification; (ii) the 3′ end is suitable for some chemical modifications; and (iii) PS linkage replacement usually has an adverse effect on improving the efficiency of genome editing. Compared to unmodified gRNAs, chemical modification of gRNAs can significantly improve editing efficiency. This is because the chemical modifications enhance gRNA tolerance to intracellular nucleases. Generally, chemically modified gRNAs show a limited increase in gene-editing efficiency compared to plasmid-expressed gRNAs. Nevertheless, chemical modification of gRNA continues to hold significant value for clinical treatment and in vitro delivery.

## 4 Cas nuclease-related design and covalent binding

**Fig. 4:**
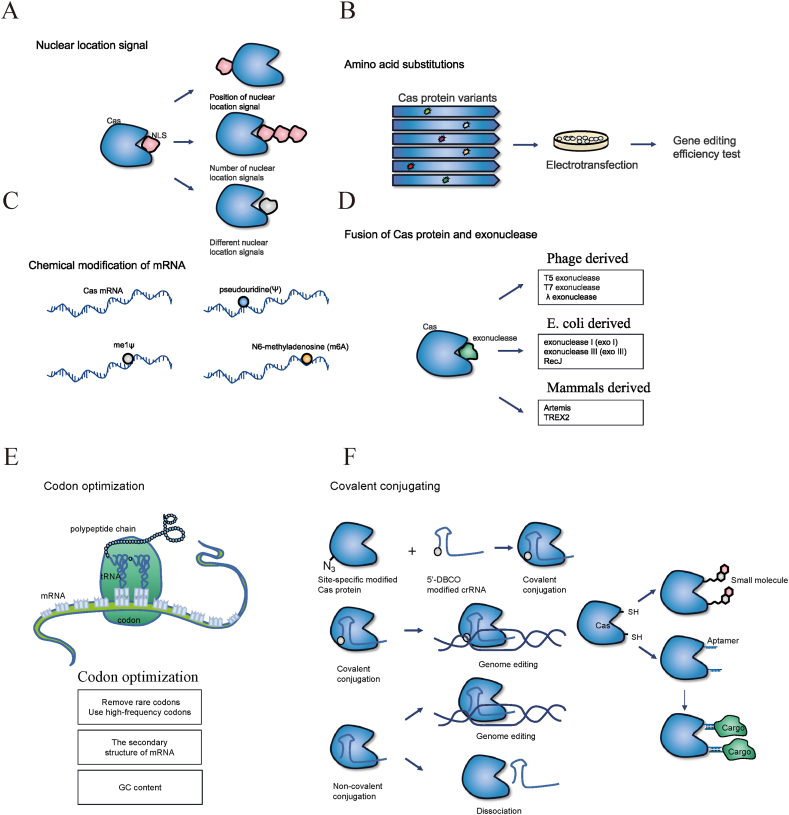
Cas-related typical operations that can improve editing efficiency. (A) Influence of NLS on editing efficiency. Factors that affect the accessibility of Cas protein to the genome include the location (N- or C-terminal), number, and type of NLS. (B) Effect of amino acid substitution in Cas protein on editing efficiency. The Cas protein variant library is generated by engineering the Cas protein, and then transfected into the cell. The editing efficiency is tested to find the Cas variant with significantly improved efficiency. (C) Effect of chemical modification of Cas mRNA on editing efficiency. Chemical modification of Cas mRNA can affect mRNA stability. Representative chemical modifications of Cas mRNA include pseudouridine (Ψ), me1ψ, and N6-methyladenosine (m6A) modification. (D) Fusion of Cas protein with exonuclease. Exonucleases can be fused to the Cas nuclease to increase incompatible overhangs. Exonucleases are generally classified according to their source. (E) Cas protein codon optimization. Codon optimization of the Cas protein coding sequence for the host is beneficial to Cas protein expression. (F) Covalent conjugation of Cas protein. Cas protein and crRNA covalent conjugation can ensure that ribonucleoprotein will not dissociate before reaching the target site, thus improving efficiency. An adapter can be added to the Cas protein to allow conjugation of other molecules. Cas: CRISPR-associated proteins; NLS: nuclear localization signal.

In addition to optimizing gRNA to improve the editing efficiency of the CRISPR/Cas system, an important aspect to improve the editing efficiency is Cas nuclease-related optimization, including nuclear localization signals (NLSs), amino acid substitutions, chemical modification of Cas mRNA, fusion expression of Cas protein and exonuclease, codon optimization, and covalent conjugation (Fig. 4).

### 4.1 Nuclear localization signal

The accessibility of the Cas protein to the genome is affected by its ability to enter the nucleus. Despite efforts to optimize the CRISPR-Cas components, intranuclear delivery needs to be promoted. The CRISPR/Cas protein reaches the target position through an NLS and then performs cleavage via gRNA. NLSs are small peptides that mediate the nuclear import of molecules. The number and location of NLSs determine the ability of CRISPR/Cas proteins to enter the nucleus and affect their functions. Previous research on SpCas9 revealed that a single SV40 NLS mediated inefficient nuclear localization [97]. To improve the efficiency of nuclear localization, different combinations of N- and C-terminal regions were compared. Two NLSs, SV40 and nucleoplamin-long NLS, fused to the C-terminus of SpCas9, AsCas12a, LbCas12a, and FnoCas12a were the most effective combinations for nuclear localization in mammalian cells and zebrafish embryos [98]. Another type of double NLSs has also shown promising results. Ten variants of the dCas12f-VPR were designed using different locations, types, and numbers of NLSs. One variant, containing N-terminal SV40 and C-terminal c-Myc NLSs, modestly enhanced gene activation [22]. In optimizing the composition and structure of the NLS of Cas12a, the efficiency of Cas12a with three NLSs (3 × NLS-NLP-cMyc-cMyc) was most effective (3 × NLS-NLP-cMyc-cMyc >2 × NLS-NLP-cMyc >2 × NLS-NLP-SV40) in human transformed cell lines and primary cells [99]. Researchers have begun to investigate the potential of additional NLSs to further enhance editing efficiency. The high-specificity eSpCas9(1.1) nuclease (eCas9.2NLS) with two additional NLSs (eCas9.4NLS) improves protein nuclear compartmentalization and enhances DSB formation. The delivery of eCas9.4NLS into muscle progenitor cells via adenovirus vector resulted in an editing efficiency up to 2.7 × higher than that of eCas9.2NLS. Additionally, at least a four-fold higher dose of eCas9.2NLS (MOI 10) is required to achieve the editing efficiency produced by eCas9.4NLS (MOI 2.5) [100]. Furthermore, the knockout efficiency of AsCas12a can be improved by increasing the number of N- or C-terminal NLSs and replacing common SV40 NLS with c-Myc NLS. The addition of six NLSs significantly improved the knockout efficiency by 4–32 times [86].

In summary, the number, type, and location of NLSs play important roles in the function of Cas nuclease. It seems that the more NLSs, the better; however, if the efficiency has reached such a high level, the addition of an NLS is not necessary. In addition, c-Myc NLS appears to have a greater proficiency for nuclear import than SV40 NLS [86,99]. An NLS at both the N- and C terminals of the Cas protein is preferable, which will generally increase the editing efficiency. If one end has no NLS, the gene-editing efficiency may be unsatisfactory [22,100].

### 4.2 Amino acid substitutions

Amino acid mutagenesis of Cas proteins can be performed by various methods to obtain a significant increase in efficiency and editing performance. Here, we present several important methods related to amino acid mutagenesis of Cas proteins including structure-guided mutagenesis, mutagenesis screening, site-directed evolution, phage-assisted evolution of Cas, and Cas fusion proteins.

(1) Structure-guided mutagenesis

Structure-guided mutagenesis is a crucial method for amino acid mutation of Cas proteins. Improving Editing Activity by Synergistic Engineering (MIDAS) robustly increased the indel efficiency of Cas12i (Cas12i2) by enhancing Cas protein interactions with PAM and ssDNA substrates. Compared to that of the WT, the efficiency of the Cas12i, Cas12b, and CasX variants of the MIDAS project were significantly improved by 137, 11, and 465-fold, respectively. Cas12i^Max^ obtained by this method has a high editing efficiency and a wide range of PAM sequences, such as NTNN, NNTN, NAAN, and NCAN, which cover 70 % of the human genome. The new Cas12i^Max^ variant, Cas12i^HiFi^, exhibits high specificity [101]. Cas12i3 exhibits significant structural similarity to the aforementioned Cas12i2. Using the reported structure of Cas12i2, researchers predicted the structure of Cas12i3. Based on this structural prediction, they identified the residues involved in the interaction between Cas12i3, crRNA, and substrate DNA. To enhance the electrostatic interactions between these residues and nucleic acids, the residues were mutated to the positively charged arginine. This led to the identification of a Cas12i3 variant with the mutations S7R, D233R, D267R, N369R, and S433R, designated as Cas-SF01. Cas-SF01 exhibited ∼3-fold increased activity in generating indel mutations compared to the wild-type [102]. Similarly, an important method for optimizing the Cas protein is to obtain Cas protein-engineered variants through structure-guided engineering. An improved engineered Cas12a (iCas12a) variant showed superior genome-editing efficiency (2–5-fold relative to WT Cas12a) at genomic sites difficult to edit using WT Cas12a [103]. To enhance the activity of native LbdCas12a, researchers utilized structure-guided protein engineering. Negatively charged residues within 10 Å of the target DNA in LbdCas12a were systematically mutated into positively charged arginines. A quadruple mutant (D156R, D235R, E292R, and D350R) of LbCas12a, called hyperCas12a, was generated. The catalytic death version, hyperdCas12a, showed significantly enhanced gene activation efficiency (approximately 60 × that of the WT protein), specifically at low concentrations of crRNA (more than 300 × that of the WT protein). The hyperdCas12a had off-target effects comparable to those of WT dCas12a. The delivery of hyperdCas12a with miniVPR and a crRNA array simultaneously activated the endogenous Oct4, Sox2, and Klf4 genes in the retina of postnatal mice and altered the differentiation of retinal progenitor cells [104]. Furthermore, the new AsCas12a variant (AsCas12a-Plus) obtained by amino acid mutation increased activity by 1.5–2.0-fold, and the specificity was also improved. By introducing the corresponding substitution into LbCas12a, LbCas12a-Plus was produced with a similar performance [105]. Additionally, conserved motifs and residues in the binding pocket of the target DNA of Cas12f were predicted, and 28 candidate amino acids were selected. Each candidate amino acid was mutated to arginine to generate a variant library of dCas12f. The D143R/T147R/K330R/E528R variants showed nearly 200-fold gene activation compared to the WT [22]. By accurately altering residues associated with the active site, structure-guided mutagenesis can significantly enhance the cleavage activity, binding affinity, or other specific functions of Cas proteins, such as broadening the PAM recognition range. A common strategy is to enhance the affinity for negatively charged nucleic acid substrates by substituting non-positively charged amino acids in Cas proteins, which interact with crRNA or dsDNA, with positively charged arginine residues, based on the 3D structure of the Cas-crRNA-dsDNA ternary complex. This affinity is primarily achieved because the amino and guanidinium groups of arginine can act as hydrogen bond donors, forming hydrogen bonds with the oxygen atoms in nucleic acids. Additionally, the positively charged arginine can form salt bridges with the negatively charged nucleic acids. These mechanisms help stabilize the interaction between arginine and nucleic acids. Specifically, tools such as protein-ligand interaction profiler (PLIP) (https://plip-tool.biotec.tu-dresden.de) can be used to analyze the residues in the Cas protein that interact with crRNA or dsDNA within the Cas-crRNA-dsDNA ternary complex, as determined via electron microscopy. The identified residues can subsequently be mutated in silico to the positively charged arginine. Afterward, PLIP is employed again to assess whether these mutations strengthen the interactions. For cases where the Cas protein structure is unknown, structural prediction tools such as SWISS-MODEL (https://swissmodel.expasy.org) or AlphaFold 3 (https://www.alphafoldserver.com) can be used to generate a model of the Cas protein. Molecular docking can subsequently be performed to construct the Cas-crRNA-dsDNA ternary complex, followed by mutational analysis based on the predicted structure.

(2) Mutagenesis screening

Gene saturation mutagenesis and screening of 422 amino acids of AsCas12f were performed using deep mutation scanning technology. The highly efficient single mutations obtained were then combined to obtain variants, such as AsCas12f-YHAM (F48Y/S188H/V232A/E316M) and AsCas12f-HKRA (I123H/D195K/D208R/V232A). Combined with gRNA modifications, these two variants show an editing efficiency comparable to that obtained with Cas9 in human cells and can be efficiently used for knock-in/knockout activity and transcription activation [82]. By constructing diverse mutation libraries, mutation screening can explore a broader sequence space, thereby uncovering beneficial mutations that are difficult to predict through rational design. Mutation screening can leverage high-throughput screening technologies to rapidly identify functional mutations from a vast array of random mutations.

(3) Site-directed evolution

The requirement for a TTTV PAM sequence hinders the widespread use of CRISPR-Cas12a nucleases. To address this limitation, researchers designed ten variants that could potentially alter or form novel PAM proximal DNA contacts. Ultimately, they developed a Cas12a variant (E174R/S542R/K548R), named enAsCas12a, which expanded the targeting range approximately seven-fold [106]. For Cas12b, wild-type BhCas12b exhibits a preference for cleaving the non-target DNA strand rather than inducing DSBs at 37 °C, thereby reducing editing efficiency. The amino acid mutation K846R/S893R/E837G yielded the BhCas12b v4 mutant. The purified BhCas12b v4 protein showed increased dsDNA cleavage activity and reduced nicked dsDNA at 37 °C and showed high specificity. In the DNMT1 locus, 101 insertion sites for Cas9 were detected via Guide-seq analysis, and 90 % of the reads failed to map to the target site, whereas no off-target sites of BhCas12b v4 were observed [23]. Site-directed evolution allows researchers to design mutations based on existing structural and functional information, thereby enhancing the functionality of Cas proteins more effectively. This approach can avoid the inefficiencies or harmful mutations that may arise from random mutagenesis.

(4) Phage-assisted evolution of Cas

Researchers employed phage-assisted continuous evolution to develop an expanded PAM SpCas9 variant (xCas9) capable of recognizing a wide range of PAM sequences, including NG, GAA, and GAT [50]. Similar approaches have been applied to a novel compact Cas9 nuclease known as SlugCas9. Researchers employed phage-assisted evolution to design a SlugCas9 variant capable of recognizing the NNG PAM sequence, thereby expanding the targeting range [107]. *Campylobacter jejuni* Cas9 (CjCas9) is another compact Cas9 nuclease with a length of only 984 amino acids. Researchers employed phage-assisted continuous directed evolution to broaden the PAM compatibility of CjCas9. The identified variant, termed evoCjCas9, primarily recognizes N_4_AH and N_5_HA PAM sequences, which occur at ten times the frequency of the canonical N_3_VRYAC PAM in the genome. Additionally, evoCjCas9 exhibits a higher nuclease activity on canonical PAMs than wild-type CjCas9 [108]. The advantages of phage-assisted evolution lie in the ability of bacteriophage to rapidly evolve and adapt within its host range, providing efficient selective pressure that facilitates the screening of Cas variants with superior performance. Moreover, phage-assisted evolution enables high-throughput screening methods, such as the screening of large-scale Cas variant libraries.

(5) Cas fusion proteins

In addition, different types of RNA-binding domains have been fused into the tip of the unique β hairpin loop near the active site of LwaCas13a to enhance the RNA binding affinity. Under various buffer conditions, the two LwaCas13a variants showed improved collateral activity and could be applied for the ultrasensitive detection (attomolar concentrations) of nucleic acids, such as the detection of SARS-CoV-2 [109]. Such variants have the potential of to enable ultrasensitive detection of a wide range of RNA targets with clinical, biological, and environmental importance.

In conclusion, while various strategies exist for inducing amino acid mutations in Cas proteins, structure-guided mutagenesis remains the most widely adopted. This method, grounded in 3D structural analysis, enables the rapid identification of critical residues to enhance interactions with crRNA or dsDNA. Numerous studies have confirmed its effectiveness. However, one limitation is that it does not allow for the convenient mutation of all amino acids. In contrast, random mutagenesis screening enables mutations across all amino acids, not limited to those directly involved in interactions with crRNA or dsDNA. This allows for indirect modulation of these interactions by altering internal protein dynamics. Moreover, mutagenesis screening is not restricted to substitutions with arginine; other amino coding acids can also be incorporated. In future, mutagenesis screening is likely to become increasingly favored owing to its broader scope, enabling the identification of efficient mutations that enhance the editing efficiency, specificity, or target range of the CRISPR/Cas system.

### 4.3 Chemical modification of Cas mRNA

The stability of mRNA is largely determined by its nucleotide sequence, which affects its secondary and tertiary structures and the accessibility of various RNA-binding proteins to mRNA. High-throughput sequencing has revealed that mRNA modification also plays an important role in regulating mRNA stability. In the MODOMICS database, >300 different RNA modifications have been identified in encoded and non-encoded RNA, including N6-methyladenosine, N6, 2′-O-dimethyladenosine, and pseudouridine (Ψ) [110,111]. Different RNA modifications have different molecular mechanisms for stabilizing RNA [112]. Despite transcribing Cas mRNA in vitro, and adding a 5′ cap and 3′ tail, the resulting mRNA remains unstable and exhibits certain immunogenicity, thus hindering its function. Substituting 25 % of uracil and cytidine with 2-thiouridine and 5-methyl-cytidine decreased mRNA binding to pattern recognition receptors such as TLR3, TLR7, TLR8, and RIG-1 in human peripheral blood mononuclear cells [113]. This led to a reduction in the innate immune response, similar to the outcome observed for Cas proteins [114]. HEK293T cells were treated with the unmodified AsCpf1, ψ- and me1ψ-modified AsCpf1 mRNA and the resulting activity of AsCpf1 mRNA modified by ψ- and me1ψ was higher than that of unmodified AsCpf1 (177 %, 168 % and 154 % respectively) [83]. Although chemical modification of Cas mRNA can improve its stability and reduce immune system recognition, this method is more expensive than gRNA chemical modification. Additionally, some modifications may negatively impact Cas mRNA function, including translation and stability. To prevent introducing additional abnormalities, it is crucial to ensure that the chemical modification does not damage normal cells.

### 4.4 Fusion expression of Cas protein and exonuclease

Once DSBs occur at the target site, the endogenous NHEJ pathway repairs the broken DNA ends. Generally, NHEJ uses short homologous DNA sequences to guide repair [115]. Short homologous DNA sequences usually exist as single-stranded overhangs at the ends of DSBs. When the overhangs are fully compatible, NHEJ will accurately repair the DSB without causing indels [116,117]. In contrast, incompatible overhangs tend to cause indels and improve knockout efficiency. Exonucleases can eliminate the protruding overhangs. Therefore, exonucleases can be fused to the Cas nuclease to increase incompatible overhangs and improve the gene knockout efficiency. Exonucleases are generally classified according to their source. They are derived from phages, such as T5 exonuclease, T7 exonuclease, and λ exonuclease; from *Escherichia coli*, such as *E. coli* exonuclease III (exoIII); and from mammals such as Artemis. *E. coli* exonuclease I (sbcB) is a 3′→5′ exonuclease. In zebrafish, the sbcB-Cas9 fusion protein showed an improved rate of mutant phenotypes and produced longer deletions than Cas9 alone [19]. In addition, the fusion of SpCas9 with the exonuclease RecJ (C9R) or GFP (C9G) led to a greater targeted mutagenesis efficiency of up to 600 % without increasing off-target effects compared to SpCas9 (C9). These two functionally enhanced fusion proteins, C9R and C9G, known as CRISPR PLUS, should be able to edit hard-to-edit genes, making it easy to obtain cells with design traits. However, fusion with GFP can also significantly improve editing efficiency. This may be because SpCas9 forms a favorable conformation after fusing with other proteins. Therefore, fusion with any other protein that improves editing efficiency may involve a hidden influencing factor, which is a change in protein conformation [18]. TREX2 is a type of 3′ exonuclease from humans that degrades DNA with 3′ protruding ends and is involved in DNA replication, recombination, and repair. The fusion of SpCas9 with TREX2 increases mutagenic efficiency by 2.5-fold [118]. Similarly, the fusion of SpCas9 with TREX2 produces Cas9 exo-endonuclease (Cas9TX) which improves the editing efficiency and reduces the occurrence of chromosome abnormalities in engineered T cells and other test cells [119]. In addition, binding of T5 exonucleases to FnCas12a improves the editing efficiency of FnCas12a in different human cell lines by 2–3 fold [120]. To reduce the impact of fusion expression on the spatial structure and sequence length of nucleases, some researchers recruited exoIII based on a coiled-coil heterodimer into the CRISPR/Cas9 system (called CCExo). This system showed an increase in deletion size (up to 40 bp) and gene knockout efficiency (a 7-fold increase compared to the WT). Further, in leukemia patient cells or animal models, the use of CCExo targeting led to the reduction or elimination of cancer [121]. Fusion of Cas proteins with exonucleases generally improves editing efficiency, and all show an increase in deletion size, which is beneficial for gene knockout and loss of function, although exonuclease fusion results in increased plasmid length. Some endogenous exonucleases play a crucial role in DNA replication and repair, acting as protectors of genome stability. Whether introducing exogenous exonucleases will disrupt the normal function of endogenous exonucleases remains unclear. Additionally, it is important to consider that the exogenous exonucleases may have toxic or unknown effects on cells. Therefore, the advantages and disadvantages must be carefully weighed before application to ensure safety and efficacy.

### 4.5 Codon optimization

The standard genetic code consists of 64 codons, of which 61 encode 20 standard amino acids. The degeneracy of codons enables the same amino acid to be encoded by multiple synonymous codons. However, synonymous codons are not used with equal frequencies. The preferential use of certain synonymous codons, a phenomenon called codon usage bias, has been widely found [[122], [123], [124]]. Codon optimization is required for efficient expression in mammals. By removing the “rare” codons and using the codons that match the expression frequency of the host-biased codons, protein expression efficiency will be greatly improved. It is also necessary to consider the combinatorial usage frequency of codons, as the combination of high- and sub-high-frequency codons will have a more potent effect. In addition, the secondary structure and GC content of mRNA have a significant influence on protein expression. Many codon optimization algorithms have been reported [[125], [126], [127]]. The advantages and disadvantages of codon optimization schemes derived from these algorithms can be analyzed. However, numerous hidden factors that are difficult to quantify and evaluate affect protein expression. Therefore, optimization schemes must be verified for the target cells to ensure satisfactory optimization results. The cytidine base editor (BE4) was optimized using IDT, GeneArt, Coller, GenScript, and IDT-GenScript codon optimization methods. Each of the latter four methods improved the editing efficiency in HEK293T cells compared to the use of the IDT codon optimization method [128].

### 4.6 Covalent conjugating

Chemical conjugation of proteins has emerged as a robust approach for constructing proteins with potential clinical applications, such as antibody-drug conjugates for cancer treatment [129,130] and antibody-DNA conjugates for diagnosis [131]. By covalently conjugating nucleases with crRNA, the ribonucleoprotein can be prevented from dissociating before reaching the target site, with improved editing efficiency. In addition, the covalent conjugation of nucleases with other favorable factors may induce mutations. The binding affinity between Cas12a and crRNA varies from a few nanometers to a few dozen nanomoles [81], which is notably lower than the range reported for Cas9 and sgRNA(K_d_ = 10 pM) [132]. Therefore, the reason for the low editing efficiency of Cas12a may be its low affinity for crRNA. A Cas12a protein, mutated by non-standard amino acids through extended genetic code, is site-specific and modified with an azide group. Uncanonical amino acid mutagenesis was utilized to generate a Cas12a protein with an azide group. This protein was called the conjugated Cas12a complex (cCas12a), which covalently conjugated the 5′ terminal-modified crRNA to the site-specific modified Cas12a. cCas12a increased the editing efficiency by 8.7 × more than the WT, when used for the preparation of quadruple-gene knockout CAR-T cells [133]. In addition, Cas9 can effectively couple molecules by replacing site-specific residues with engineered cysteine without losing Cas9 activity. A short oligonucleotide handle “adapter” was designed because many possible conjugates (such as single-stranded oligo DNA nucleotides) are extremely expensive for direct thiol-maleimide conjugation. This handle adapter is chemically connected to Cas9 through thiol-maleimide and uses base pairs to anchor any molecule containing nucleic acid. Using this method, INS-1E (a β cell line) was successfully engineered to secrete insulin to achieve glucose-dependent secretion of the protective immunomodulator interleukin-10 [134]. However, the use of chemically modified Cas proteins for covalent binding may be limited in certain applications that require the genetic expression of CRISPR components, as these proteins are recombinantly expressed and purified.

## 5 CRISPR/Cas tools through functional domain integration

### 5.1 Base editors and their optimization

Base editors (BEs) can precisely induce point mutations without DSBs or donor DNA templates. Beyond cytosine base editors (CBEs) using cytidine deaminase, adenosine base editors (ABEs) and other types such as CGBEs exist. Researchers have optimized BEs, creating high-performance variants. They have also created BEs that do not rely on deaminase.

(1) Cytosine base editor

The first-generation base editor (BE1) was developed by fusing dCas9 with rat APOBEC1, achieving 0.8 %–7.7 % efficiency. Adding a uracil glycosylase inhibitor (UGI) to BE1 reduced uracil loss, creating the second-generation base editor (BE2). Replacing dCas9 with nCas9 in BE2 led to the third-generation base editor (BE3), which demonstrated significantly higher editing efficiency than BE1 and BE2 [135]. David Liu et al. further improved BE3 by increasing the number of UGIs and optimizing the linker, resulting in the fourth-generation base editor (BE4) with enhanced efficiency and purity [136]. Subsequent efforts by various research groups have optimized CBEs to enhance editing efficiency by improving DNA accessibility, reducing uracil intermediate loss, refining deaminase-Cas fusion, and enhancing target interactions. Fusion of BEs with pioneer factor SOX increased the editing efficiency of both GBEs and CBEs by enhancing chromatin accessibility [137]. To counteract the hydrolysis of uracil-by-uracil DNA glycosylase (UDG), which lowers the editing efficiency, an antisense RNA-enhanced CRISPR/Cas9 base editing method (asRNA-BE) was developed. This method transiently disrupts UDG expression, boosting editing efficiency by 2.8–65.8-fold [138]. Embedding deaminase at various positions within SaCas9, particularly between amino acids 693 and 694, resulted in Sa-CBE-693. This variant demonstrated higher editing efficiency, a significantly expanded editing window of 2–18 bases, and reduced off-target effects [139]. Engineering nCas9 to enhance target interactions has been demonstrated to be an effective strategy for improving base editing. In AncBE4max, five residues in the RuvC domain (D54, N980, T1314, N1317, A1322), which are spatially proximate to the DNA NTS single-strand region, were mutated to arginine and combined. The control AncBE4max showed a median efficiency of 15.5 %, whereas the top three double-substitution variants achieved efficiencies of 37.3 % (S55R-N1317R), 41 % (S55R-A1322R), and 38.9 % (N1317R-A1322R) [140]. Other variants have been reported to enhance editing efficiency and also broaden the editing scope or reduce off-target effects. These include Target-AID [141], hA3A-BE3 [142], HF-BE3 [143], BE4max [128], tBE [144], TadCBE [145], SpRY-CBE [146], CjBEmax [147], ThermoBE4 [148], nNme2-CBE [149], A3A-PBE [150], DdCBE [151], each with unique advantages and performance characteristics. CBEs have been applied in mapping mutation landscapes [152], regulating gene expression [153,154], and constructing human disease models [155,156]. Additionally, CBEs have been applied in agricultural biological breeding, such as for improving livestock traits [157,158] and in various plant applications [159,160].

(2) Adenine base editor

After developing CBEs, David Liu et al. further advanced adenine base editing by evolving Escherichia coli deaminase TadA through seven rounds of protein engineering, resulting in ABE7.10. TadA deaminase converts adenine (A) to inosine (I), recognized as guanine (G) during cellular repair processes, thereby completing the A-to-G conversion [161].

Similar to the optimization and development process of CBEs, ABEs have also undergone a series of refinements. In 2020, David Liu et al. developed the highly efficient ABE8e variant, which showed over 1100-fold increase in deamination activity compared to ABE7.10, leading to significantly enhanced editing efficiency [162]. Subsequently, researchers further improved ABE performance through modifications such as enhancing Tad deaminase, optimizing deaminase-Cas fusion, and utilizing histone deacetylase inhibitors. In 2022, Li et al. reported that ABE8e with the N108Q mutation reduced bystander editing and introduced an additional L145T mutation to create ABE9, narrowing its activity window to 1–2 bp [163]. Similarly, engineering NG-ABE8e led to NG-ABE9e with nine mutations, showing over a seven-fold reduction in bystander editing compared to NG-ABE8e at certain sites [164]. Yao et al. developed SpRY-ABE8e^F148A^ by combining SpRY nucleases lacking a PAM with TadA-8e deaminase mutated at F148A, achieving a narrower editing range and enhanced A-to-G editing efficiency at most NR/YN PAM sites [165]. Nme2ABE8e, a compact adenine base editor with a smaller N4CC PAM, enhances editing efficiency significantly by positioning the deaminase domain closer to the non-target strand between amino acids 797 and 798 of Nme2ABE8e [166]. The addition of histone deacetylase inhibitor, romidepsin, increases protein expression and target accessibility, boosting base editing efficiency 4.9-fold [167]. Other optimized variants, such as ABE8e (WQ) [168], ABE9 [169], cjABE8e [170], SpRY-ABE [146], SpRY-ABE8e(F148A) [171], igRNA-ABE [172], LbABE8e-G532R/K595R [173], PhieABE [174], STUminiABE [175], have expanded the toolkit for single base editing. These ABE systems, such as CBEs, find applications in disease therapy [[176], [177], [178]], animal disease model creation [179], and agricultural biological breeding [180,181].

(3) Other base editors

CBE and ABE systems are limited to base conversion rather than base transversion. In the CBE system, after the deamination of C to form U, it may be excised by the cellular endogenous uracil glycosylase (UNG), resulting in an apurinic/apyrimidinic (AP) site, which has the potential to randomly introduce a base [135]. Based on this, researchers attempted to develop single-base editors capable of achieving base transversion by leveraging endogenous cellular repair pathways. Two research teams successfully replaced UGI with UNG to develop GBE and CGBE single-base editing systems, achieving C to G transversion [182,183]. Through engineering TadA-8e, researchers have also achieved C to G transversion, leading to the development of the Td-CGBE single-base editing system [184]. Researchers additionally engineered N-methylpurine DNA glycosylase (MPG). They fused it with ABE8e to develop AYBE, a single-base editing tool capable of achieving A to C or A to T transversions [185]. Despite enabling base transversions, systems such as GBE, CGBE, Td-CGBE, and AYBE require further enhancements in editing efficiency and precision compared to CBE and ABE systems.

### 5.2 Primer editors and their optimization

While BEs can achieve a variety of base conversions, they are limited in their ability to perform all types of conversions, including base insertions and deletions. In 2019, David Liu et al. introduced the PE system, which facilitates all 12 types of base conversions as well as multiple base insertions and deletions without the need for DSBs [186].

The PE system consists of two components: an effector protein formed by the fusion of nCas9 (H840A) with reverse transcriptase (RT) and pegRNA. The pegRNA comprises a primer binding site (PBS) sequence and a reverse transcriptase template (RT template). Under the guidance of pegRNA, nCas9 induces a cut 3 nt upstream of the PAM sequence, releasing a ssDNA that pairs with PBS. Subsequently, reverse transcription occurs using the RT template to synthesize new DNA. The target editing sequence on the pegRNA is then transferred to the non-target DNA strand and subsequently integrated into the genome through DNA repair, replacing the originally cleaved DNA sequence. To enhance editing efficiency, David Liu et al. developed several PE variants: PE1, PE2, PE3, and PE3b. The PE2 system involved screening for M-MLV mutants with five-point mutations (D200N, L603W, T330P, T306K, and W313F), resulting in an increase in editing efficiency by 1.6–5.1-fold. The PE3 system further enhanced editing efficiency by 1.5–4.2-fold through the introduction of nicking sgRNAs, albeit with some induced indels. PE3b, while maintaining editing efficiency, reduces indels caused by nicking sgRNAs [186]. While PE presents powerful functionalities and high potential, its editing efficiency remains relatively low. To address this limitation, researchers have enhanced the PE editing efficiency through engineered pegRNAs and engineered nCas9-RT fusion proteins. These diverse strategies have significantly improved the editing efficiency of PE to varying extents, thus advancing the development of PE editing tools.

(1) Engineering pegRNA

In the PE system, pegRNA is pivotal. Researchers observed that pegRNA constructs can self-circularize, leading to the development of the ePE system through the introduction of the Csy4. This innovation reduces self-circularization, thereby enhancing PE efficiency [187]. Further improvements were made by introducing mutations to stabilize the pegRNA structure [188]. Additionally, the 3′ extension of pegRNA, unprotected by the nCas9-RT fusion protein, is susceptible to degradation by cellular nucleases. To address this, evopreQ1 was appended to the 3′ end of pegRNA, resulting in the epegRNA and a subsequent 3–4-fold increase in prime editing efficiency [189]. Several studies have demonstrated that incorporating secondary or tertiary structural motifs at the ends of pegRNAs can enhance their stability. Examples include the use of G-quadruplex structures [190] and aptamer motifs (MS2) [191]. These engineered pegRNAs have shown significant improvements in PE editing efficiency.

(2) Engineering nCas9-RT fusion protein

The engineering of nCas9-RT fusion proteins has significantly enhanced the editing efficiency of PE. Studies have shown that converting nCas9 to Cas9 can substantially improve precise editing efficiency, albeit with a marked increase in indel formation [192]. nCas9 (H840A) was found to cleave both DNA strands, leading to unwanted DSBs. To mitigate this, researchers introduced an N854A mutation into nCas9 (H840A). When this variant was employed in the PE system alongside engineered pegRNA (ePE3), the nCas9 variant (H840A + N854A) significantly increased the frequency of accurate edits [193]. Through the engineering of various RT enzymes and protein modifications, David Liu et al. advanced a range of PE tools, including PE6a-g, significantly bolstering the efficiency [194]. Researchers optimized the PE2 expression construct by refining the nuclear localization signal, leading to the creation of the PE∗ and PEmax systems, which significantly improved editing efficiency [195,196].

Moreover, Gerald Schwank et al. have harnessed the OrthoRep platform, a yeast-based directed protein evolution system, to identify two mutations that significantly enhance PE editing efficiency, namely A259D in nCas9 and K445T in M-MLVRT. These two mutations were combined to form the PE_Y18 variant. Delivery of PE_Y18 to mammalian cell lines as DNA, mRNA, or RNP resulted in a 3.5-fold increase in editing efficiency compared to PEmax [197]. Concurrently, Zong Yuan et al. developed an enhanced plant PE editor for hexaploid wheat, termed ePPEplus, by incorporating a V223A substitution into the RT within the ePPEmax∗ structure. Notably, ePPEplus demonstrated an average efficiency increase of 33.0-fold and 6.4-fold compared to the original PPE and ePPE, respectively [198]. Additionally, the introduction of nucleases to facilitate the degradation of the genome's native 3′ flap DNA strand can further boost the editing efficiency of PE [[199], [200], [201]]. The efficiency of PE has been improved to some extent through the engineering of the nCas9-RT fusion protein described above.

### 5.3 Tools regulating gene expression

(1) CRISPR activation

Current CRISPR activation (CRISPRa) systems can be categorized into two types: one involves directly fusing transcriptional activation domains to the dCas protein, and the other consists of recruiting transcriptional activation domains to the dCas protein or the sgRNA scaffold.

The first CRISPRa system developed involved adding VP64 to dSpCas9; however, this led to only low-intensity upregulation [202]. To enhance transcriptional regulation efficacy, the VP64-p65-Rta (VPR) fusion was designed to be fused with dCas9, achieving RNA expression levels 22 to 320 times higher than those achieved with dCas9-VP64 in mammalian cells [203]. Subsequently, VPH was developed, combining four VP48 repeats, p65, and HSF1 [204]. Another reported effector, miniVPR, is a truncated version with p65 and Rta domains [205]. However, the number of directly fused proteins remains limited, which constrains regulatory efficiency. Thus, recruiting more transcriptional effectors to the promoter region may enhance transcriptional activation, leading to the development of the second category of systems. The synergistic activation mediator (SAM) system utilizes a basic dSpCas9-VP64 fusion protein, where the sgRNA hairpin structure is modified to include two aptamers, each carrying binding sites for two MS2 proteins. The MS2-p65-HSF1 fusion protein can be recruited to the MS2 aptamers, theoretically delivering four copies of p65-HSF1 to each dCas9-VP64 complex, thereby achieving robust transcriptional activation [88]. In addition to the SAM system, another important system is the SunTag system. In the system, SunTag is fused to dCas9. SunTag, a polypeptide chain containing multiple epitopes, can recruit single-chain fragments variable (scFv) derived from antibodies known as GCN4, which are linked to VP64. This approach allows multiple copies of VP64 to be recruited to a single dCas9 protein, resulting in more effective upregulation of target genes compared to using dCas9-VP64 alone [206,207].

(2) CRISPR interference

The CRISPRi system initially used only dCas9 to sterically block RNA polymerase, thereby interfering with the transcription of target genes [208]. Later, the inhibitory effect was enhanced by linking the KOX1 krüppel-associated box (KRAB) to dCas9 [209]. KRAB domains promote the tri-methylation of histone H3 lysine 9 (H3K9), resulting in robust transcriptional repression [210]. Studies have shown that the tandem fusion of multiple transcriptional regulators with dCas9 can further increase CRISPRi knockdown efficiency. Researchers tested over 20 different effector domains and identified six that demonstrated strong inhibitory effects. Combining these six inhibitory factors with KOX1 KRAB, they determined that the methyl-CpG-binding protein 2 (MeCP2)-KRAB domain produced the most potent transcriptional interference [211]. Subsequently, researchers tested 57 different KRAB domains and identified the ZIM3 KRAB domain as more effective than KOX1 KRAB-MeCP2. In addition to its potency, ZIM3 KRAB is smaller than the KOX1 KRAB-MeCP2 construct [212]. Alternatively, gene expression can be suppressed by fusing dCas9 with DNA methyltransferases (DNMTs) or histone deacetylases (HDACs) [213,214].

## 6 Compact RNA-guided nucleases and Cas12k

### 6.1 Cas12f

In 2018, Harrington et al. discovered a CRISPR-Cas system containing Cas14 (later renamed Cas12f) in uncultivated archaea [25]. Sequence databases identified Cas12f as belonging to the V-F subtype within class 2 CRISPR-Cas systems. Cas12f cleaves double-stranded DNA targets in an “asymmetric homodimer” manner guided by sgRNA and dependent on a PAM sequence. In contrast, recognition of ssDNA substrates does not require a PAM target sequence and exhibits targeted cleavage activity against ssDNA [215,216]. Here, we introduce engineered versions based on the Un1Cas12f1 (529 amino acids) and AsCas12f (422 amino acids). A highly efficient miniaturized Cas system (CasMINI) was developed from Un1Cas12f1 system through guide RNA and protein engineering. CasMINI is less than half the size of SpCas9. CasMINI has been demonstrated to drive gene activation levels thousands of times higher and allows powerful base editing and gene-editing capabilities [22]. By redesigning five sites in the natural guide RNA of Un1Cas12f1: the 5′ terminus of the trans-activating CRISPR RNA (tracrRNA), the tracrRNA–crRNA complementary region, a penta (uridinylate) sequence, the 3′ terminus of the crRNA and a disordered stem 2 region in the tracrRNA, a synergistic effect of these optimizations has been achieved. This has resulted in an 867-fold increase in the average insertion-deletion frequency. This variant is referred to as Un1Cas12f1_ge4.1 [217]. However, through protein and sgRNA engineering, enhanced variants of OsCas12f1 (enOsCas12f1) and enRhCas12f1 were generated, featuring 5′-TTN and 5′-CCD (D = non-C) PAM recognition sequences, respectively. Compared to the engineered variant Un1Cas12f1 (Un1Cas12f1_ge4.1), these variants exhibit higher editing efficiency and broader PAM compatibility [218]. In addition to Un1Cas12f1, Wu et al. engineered enAsCas12f, which exhibits 11.3-fold higher efficiency than AsCas12f. It achieves up to 69.8 % insertion and deletion of DNA segments at specific genomic loci while demonstrating minimal off-target editing activity [219]. Combined with deep mutational scanning and structural design, two active enhanced variants of AsCas12f (enAsCas12f) were successfully generated. Importantly, these enAsCas12f variants exhibit genome-editing activity comparable to that of SpCas9 and AsCas12a in human cells [82]. The newly designed AsCas12f system may serve as a promising platform for genome editing.

### 6.2 IscB

IscB, a nuclease encoded by the IS200/IS605 transposon family, is posited as a potential ancestor of Cas9. Forming a ribonucleoprotein complex with its homologous ωRNA, IscB can cleave double-stranded DNA targets complementary to the ωRNA guide sequence. While IscB shares RuvC and HNH nuclease domains with Cas9, it is markedly smaller, primarily owing to the absence of the α-helical nucleic acid recognition lobe [220]. The size of IscB is less than half that of Cas9, making it more suitable for in vivo delivery. Nevertheless, its relatively poor editing efficiency in eukaryotic cells restricts its application in vivo. By engineering OgeuIscB (IscB from the human gut metagenome) and its ωRNA, a highly efficient mammalian IscB system, termed enIscB, was developed. The fusion of enIscB with T5 exonuclease demonstrated target efficiencies comparable to those of SpCas9. Additionally, the creation of a miniature IscB-derived base editor (miBE) was achieved by fusing cytidine or adenosine deaminases with enIscB nickase, resulting in robust editing efficiencies of up to 92 % for inducing DNA base conversions [221]. The development of IscB has expanded our miniaturized CRISPR toolbox for genome editing.

### 6.3 TnpB

TnpB is another nuclease encoded by the IS200/IS605 transposon family. TnpB provides only a minimal core structural domain that performs the CRISPR-Cas12 family function. Therefore, the CRISPR-Cas12 system found in prokaryotes is believed to have evolved from TnpB by inserting an additional structural domain [222]. The ISDra2-TnpB protein from *Deinococcus radiodurans* has demonstrated efficient DNA cleavage adjacent to the 5′-TTGAT transposase-associated motif (TAM) in HEK293T cells [223]. Researchers further generated a truncated supermini TnpB editor (<400 amino acids) by shortening the C-terminal domain of ISDra2-TnpB, demonstrating effective gene editing in mammalian cells and mice [224]. Concurrently, studies have optimized the TnpB-ωRNA system through stepwise engineering to identify ωRNA variants with shorter sequence lengths and higher gene-editing activity. Importantly, optimized TnpB-ωRNA systems have been shown to effectively deliver corrections of disease phenotypes in a tyrosinemia mouse model following a single AAV injection [225]. In addition to those originating from the IS200/IS605 transposase family, recent studies have reported an ultra-compact IS607 TnpB protein that interacts with the conserved RAGATH-18-derived RNA (reRNA). The IS607 TnpB system exhibits potent dsDNA interference activity in both bacterial and human cells [226]. This demonstrates the tremendous potential of the system for applications in gene editing.

### 6.4 Fanzor

Fanzors are the eukaryotic homologs of prokaryotic TnpB proteins [227]. In eukaryotes, TnpB homologs are found in two distinct forms: Fanzor1s and Fanzor2s. Research has demonstrated that both Fanzor1s and Fanzor2s trace their origins to a single lineage of IS607 TnpBs. The evolutionary sequence progresses from IS607 TnpBs to Fanzor2s, and subsequently from Fanzor2s to Fanzor1s [228]. Fanzors and ωRNA form RNP complexes that mediate programmable RNA-guided DNA cleavage in a target- and TAM-specific manner. Enhancements in SpuFz1 activity were achieved through a 275-nt extension of the 5′ sequence or the addition of an MS2 stem-loop to the 5′ end of the ωRNA scaffold. The introduction of the C310R/D487K/T513K mutation combination further enhanced the editing efficiency, highlighting the potential of Fanzors as a genome-editing tool [229].

### 6.5 Cas12k

Cas12k is the effector protein of the type V-K CRISPR-Cas system, identified exclusively in cyanobacteria. Structurally, Cas12k is similar to other miniature Cas12 proteins, particularly Cas12f, but it lacks nuclease activity. Cas12k binds to target dsDNA in an RNA-guided and PAM-dependent manner. Cas12k operates synergistically with Tn7-like transposons, where the CRISPR effector complex directs the Tn7-like transposase to the target site, enabling the insertion of exogenous DNA fragments 60–66 bp downstream of the target DNA PAM sequence. This RNA-guided DNA transposition occurs with frequencies of up to 80 % [230]. While ShCAST (*Scytonema hofmannii* CRISPR-associated transposase) enables the integration of DNA fragments up to approximately 10 kb into *Escherichia coli*, efficiency remains suboptimal for fragments larger than 5 kb. A multifunctional genetic engineering tool based on Cas12k, termed C12KGET, has been developed, achieving 100 % integration efficiency of heterologous DNA fragments up to 10 kb without the need for selection markers [231]. To facilitate efficient integration of ultra-long DNA sequences into bacterial chromosomes, an optimized ShCAST system has been devised, achieving approximately 100 % efficiency in transposing 30 kb DNA sequences onto bacterial chromosomes [232]. To further improve integration product purity and genome-wide specificity, researchers developed the HE-assisted Large-sequence Integrating CAST-compleX (HELIX). HELIX enables cut-and-paste DNA insertion with up to 99.4 % simple insertion product purity while maintaining robust integration efficiencies at genomic targets [233]. The above work significantly expands the application scope and flexibility of Cas12k-based genetic engineering technologies.

## 7 Efforts to improve specificity

Despite improvements in editing efficiency through engineering modifications, off-target effects may constrain the application of highly efficient editing systems. This paper focuses on strategies to enhance the specificity of CRISPR/Cas systems through modifications of gRNA and Cas proteins.

Studies have shown that the target specificity of gRNA-Cas9 is primarily determined by the seed sequence located 10–12 bp upstream of the PAM motif at the 3′ end of the sgRNA. High homology between off-target sequences and the sgRNA seed region typically results in off-target effects [234,235]. Additionally, by controlling the number of base mismatches between the sgRNA and the target DNA sequence, it is possible to design sgRNAs with high cutting efficiency and reduced off-target effects [236]. Researchers have also identified that the length and composition of the sgRNA significantly influence its specificity and efficiency. For instance, truncated sgRNAs with shorter spacers (17–18 nucleotides) can reduce off-target effects by more than 5000-fold without compromising targeting efficiency [237]. In another study, 17 nucleotide sgRNAs exhibited reduced off-target effects while maintaining moderate editing efficiency in certain cell lines, indicating the need for a careful balance between targeting and off-target activity when using truncated sgRNAs [238]. Additionally, using chimeric DNA-RNA as guide RNA enhanced target specificity by an average of 2.8-fold [84]. Although these modifications to gRNA have effectively improved specificity, off-target effects have not been eliminated, necessitating further improvements in Cas proteins or other aspects to enhance specificity.

Strategies to reduce off-target effects include reducing the amount of active Cas9 in cells [239], utilizing Cas9 nickase mutants to create paired single-strand DNA nicks [240], and employing catalytically inactive Cas9 nucleases fused to FokI nuclease domains [74]. Although each method reduces off-target mutagenesis, they have limitations: reducing Cas9 levels lowers targeting efficiency, and dual nicking requires the simultaneous delivery of two guide RNAs. Among these strategies, direct or indirect engineering of Cas proteins has shown more significant improvements in Cas specificity. Zhang et al. developed two Cas9 variants, eSpCas9(1.0) and eSpCas9(1.1), which reduce off-target effects while maintaining robust target cleavage [241]. Similarly, disrupting direct hydrogen bonds between SpCas9 and the phosphate backbone of the target DNA strand resulted in the high-fidelity Cas9-HF1, which exhibits target cleavage efficiency comparable to that of SpCas9 but with significantly reduced off-target activity [242]. Although these variants offer higher specificity, some off-target cleavage still occurs. Researchers further screened a library of REC3 domain variants, and developed another highly specific SpCas9 variant called “evoCas9”, which demonstrates significantly higher fidelity than both eSpCas9(1.1) and Cas9-HF1 [243]. For in vivo editing, smaller systems are better suited for AAV vector-based delivery. *Staphylococcus aureus* Cas9 (SaCas9) is notably smaller than SpCas9 (1053 vs1368 amino acids). Researchers developed a SaCas9 variant (SaCas9-HF) by modifying residues in close polar contacts with the target DNA strand backbone in the PAM-distal region. This variant exhibits high genome-wide specificity in human cells without compromising targeting efficiency [244]. Additionally, a directed evolution screening system led to the development of another high-fidelity SaCas9 variant (efSaCas9). Targeted deep sequencing analysis showed that efSaCas9 significantly reduced off-target effects by approximately 2 to 93-fold compared to wild-type [245]. A parallel comparison using a fluorescence reporter system and targeted deep sequencing in human cells indicated that efSaCas9 shows higher cleavage activity and fidelity at most endogenous sites compared to SaCas9-HF [246]. Similar strategies were extended to the recently developed Cas12i system. By introducing arginine mutations in the RuvC-II domain of the engineered Cas12i variant Cas-SF01, the D876R mutation was identified, leading to the development of Cas-SF01^HiFi^ [102]. While these modifications reduce off-target effects, they also often reduce editing efficiency. Therefore, despite successfully identifying several high-fidelity Cas variants, they are far from perfect.

## 8 Conclusions

Gene editing technology has developed rapidly over the past decade. As a tool for gene manipulation, the most important scientific breakthrough in the past half-century, the CRISPR/Cas system has profoundly changed basic biological research, diagnosis, and treatment of diseases, and animal and plant breeding in agriculture. With the unremitting efforts of scientists, the gene editing toolbox has expanded greatly. However, for better application in various experimental and clinical settings, it is important to improve the editing efficiency of these CRISPR/Cas systems, which are accompanied by low editing efficiency and excellent performance in other aspects. In the future, in addition to improving the editing efficiency, it may be necessary to focus on the precision of gene editing and the delivery of RNA to organs and tissues. Gene editing technology is expected to become safer and more efficient in the future.

## Funding

This study was supported by the Chinese Universities Scientific Fund (2022TC025), Pinduoduo-10.13039/501100002365China Agricultural University Research Fund (PC2023A01004), and Great Projects of Agricultural Biotechnology Breeding in China (2022ZD04013).

## Ethics statement

No ethical approval was required as this study did not involve human participants or laboratory animals.

## Data availability statement

No data was used for the research described in the article.

## CRediT authorship contribution statement

**Linli Wang:** Writing – review & editing, Writing – original draft. **Hongbing Han:** Writing – review & editing, Supervision, Funding acquisition, Conceptualization.

## Declaration of competing interest

The author declares that no competing financial interests.

## References

[1] Jinek M., Jiang F., Taylor D.W. (2014). Structures of Cas9 endonucleases reveal RNA-mediated conformational activation. Science.

[2] Barrangou R., Fremaux C., Deveau H. (2007). CRISPR provides acquired resistance against viruses in prokaryotes. Science.

[3] Stadtmauer E.A., Fraietta J.A., Davis M.M. (2020). CRISPR-engineered T cells in patients with refractory cancer. Science.

[4] Larson R.C., Kann M.C., Bailey S.R. (2022). CAR T cell killing requires the IFNgammaR pathway in solid but not liquid tumours. Nature.

[5] Wang D., Chen L., Li C. (2022). CRISPR/Cas9 delivery by NIR-responsive biomimetic nanoparticles for targeted HBV therapy. J. Nanobiotechnol..

[6] Villiger L., Grisch-Chan H.M., Lindsay H. (2018). Treatment of a metabolic liver disease by in vivo genome base editing in adult mice. Nat. Med..

[7] Luo N., Li J., Chen Y. (2021). Hepatic stellate cell reprogramming via exosome-mediated CRISPR/dCas9-VP64 delivery. Drug Deliv..

[8] Zhang X., Xu C., Gao S. (2019). CRISPR/Cas9 delivery mediated with hydroxyl-rich nanosystems for gene editing in aorta. Adv. Sci..

[9] Schoger E., Carroll K.J., Iyer L.M. (2020). CRISPR-mediated activation of endogenous gene expression in the postnatal heart. Circ. Res..

[10] Broughton J.P., Deng X., Yu G. (2020). CRISPR-Cas12-based detection of SARS-CoV-2. Nat. Biotechnol..

[11] Joung J., Ladha A., Saito M. (2020). Detection of SARS-CoV-2 with SHERLOCK one-pot testing. N. Engl. J. Med..

[12] Xu K., Zhou Y., Mu Y. (2020). CD163 and pAPN double-knockout pigs are resistant to PRRSV and TGEV and exhibit decreased susceptibility to PDCoV while maintaining normal production performance. Elife.

[13] Gao Y., Wu H., Wang Y. (2017). Single Cas9 nickase induced generation of NRAMP1 knockin cattle with reduced off-target effects. Genome Biol..

[14] Zhu X.X., Zhan Q.M., Wei Y.Y. (2020). CRISPR/Cas9-mediated MSTN disruption accelerates the growth of Chinese Bama pigs. Reprod. Domest. Anim..

[15] Wang K., Ouyang H., Xie Z. (2015). Efficient generation of myostatin mutations in pigs using the CRISPR/Cas9 system. Sci. Rep..

[16] Lv Q., Yuan L., Deng J. (2016). Efficient generation of myostatin gene mutated rabbit by CRISPR/Cas9. Sci. Rep..

[17] Wang X., Yu H., Lei A. (2015). Generation of gene-modified goats targeting MSTN and FGF5 via zygote injection of CRISPR/Cas9 system. Sci. Rep..

[18] Park J., Yoon J., Kwon D. (2021). Enhanced genome editing efficiency of CRISPR PLUS: Cas9 chimeric fusion proteins. Sci. Rep..

[19] Clements T.P., Tandon B., Lintel H.A. (2017). Rice CRISPR: rapidly increased cut ends by an exonuclease Cas9 fusion in zebrafish. Genesis.

[20] Zhang H., Li Z., Xiao R. (2020). Mechanisms for target recognition and cleavage by the Cas12i RNA-guided endonuclease. Nat. Struct. Mol. Biol..

[21] Özcan A., Krajeski R., Ioannidi E. (2021). Programmable RNA targeting with the single-protein CRISPR effector Cas7-11. Nature.

[22] Xu X., Chemparathy A., Zeng L. (2021). Engineered miniature CRISPR-Cas system for mammalian genome regulation and editing. Mol. Cell.

[23] Strecker J., Jones S., Koopal B. (2019). Engineering of CRISPR-Cas12b for human genome editing. Nat. Commun..

[24] Liu J.J., Orlova N., Oakes B.L. (2019). CasX enzymes comprise a distinct family of RNA-guided genome editors. Nature.

[25] Harrington L.B., Burstein D., Chen J.S. (2018). Programmed DNA destruction by miniature CRISPR-Cas14 enzymes. Science.

[26] Dmytrenko O., Neumann G.C., Hallmark T. (2023). Cas12a2 elicits abortive infection through RNA-triggered destruction of dsDNA. Nature.

[27] Zhang B., Luo D., Li Y. (2021). Mechanistic insights into the R-loop formation and cleavage in CRISPR-Cas12i1. Nat. Commun..

[28] van Beljouw S.P.B., Haagsma A.C., Rodriguez-Molina A. (2021). The gRAMP CRISPR-Cas effector is an RNA endonuclease complexed with a caspase-like peptidase. Science.

[29] Teng F., Cui T., Feng G. (2018). Repurposing CRISPR-Cas12b for mammalian genome engineering. Cell Discov.

[30] Schuler G., Hu C., Ke A. (2022). Structural basis for RNA-guided DNA cleavage by IscB-omegaRNA and mechanistic comparison with Cas9. Science.

[31] McGaw C., Garrity A.J., Munoz G.Z. (2022). Engineered Cas12i2 is a versatile high-efficiency platform for therapeutic genome editing. Nat. Commun..

[32] Kleinstiver B.P., Tsai S.Q., Prew M.S. (2016). Genome-wide specificities of CRISPR-Cas Cpf1 nucleases in human cells. Nat. Biotechnol..

[33] Kim D., Kim J., Hur J.K. (2016). Genome-wide analysis reveals specificities of Cpf1 endonucleases in human cells. Nat. Biotechnol..

[34] Pausch P., Al-Shayeb B., Bisom-Rapp E. (2020). CRISPR-CasΦ from huge phages is a hypercompact genome editor. Science.

[35] Carabias A., Fuglsang A., Temperini P. (2021). Structure of the mini-RNA-guided endonuclease CRISPR-Cas12j3. Nat. Commun..

[36] Zetsche B., Gootenberg J.S., Abudayyeh O.O. (2015). Cpf1 is a single RNA-guided endonuclease of a class 2 CRISPR-Cas system. Cell.

[37] Makarova K.S., Wolf Y.I., Iranzo J. (2020). Evolutionary classification of CRISPR-Cas systems: a burst of class 2 and derived variants. Nat. Rev. Microbiol..

[38] Tan R., Krueger R.K., Gramelspacher M.J. (2022). Cas11 enables genome engineering in human cells with compact CRISPR-Cas3 systems. Mol. Cell.

[39] Csorgo B., Leon L.M., Chau-Ly I.J. (2020). A compact Cascade-Cas3 system for targeted genome engineering. Nat. Methods.

[40] Xiao Y., Luo M., Hayes R.P. (2017). Structure basis for directional R-loop formation and substrate handover mechanisms in type I CRISPR-Cas system. Cell.

[41] Morisaka H., Yoshimi K., Okuzaki Y. (2019). CRISPR-Cas3 induces broad and unidirectional genome editing in human cells. Nat. Commun..

[42] Jiang F., Doudna J.A. (2017). CRISPR-Cas9 structures and mechanisms. Annu. Rev. Biophys..

[43] Nishimasu H., Ran F.A., Hsu P.D. (2014). Crystal structure of Cas9 in complex with guide RNA and target DNA. Cell.

[44] Kim H.K., Song M., Lee J. (2017). In vivo high-throughput profiling of CRISPR-Cpf1 activity. Nat. Methods.

[45] Yamano T., Nishimasu H., Zetsche B. (2016). Crystal structure of Cpf1 in complex with guide RNA and target DNA. Cell.

[46] Abudayyeh O.O., Gootenberg J.S., Konermann S. (2016). C2c2 is a single-component programmable RNA-guided RNA-targeting CRISPR effector. Science.

[47] Liu L., Li X., Ma J. (2017). The molecular architecture for RNA-guided RNA cleavage by Cas13a. Cell.

[48] Xiao Y., Luo M., Dolan A.E. (2018). Structure basis for RNA-guided DNA degradation by cascade and Cas3. Science.

[49] Jinek M., Chylinski K., Fonfara I. (2012). A programmable dual-RNA-guided DNA endonuclease in adaptive bacterial immunity. Science.

[50] Hu J.H., Miller S.M., Geurts M.H. (2018). Evolved Cas9 variants with broad PAM compatibility and high DNA specificity. Nature.

[51] Christie K.A., Guo J.A., Silverstein R.A. (2023). Precise DNA cleavage using CRISPR-SpRYgests. Nat. Biotechnol..

[52] Huang T.P., Heins Z.J., Miller S.M. (2023). High-throughput continuous evolution of compact Cas9 variants targeting single-nucleotide-pyrimidine PAMs. Nat. Biotechnol..

[53] Paul B., Montoya G. (2020). CRISPR-Cas12a: functional overview and applications. Biomed. J..

[54] Kellner M.J., Koob J.G., Gootenberg J.S. (2019). SHERLOCK: nucleic acid detection with CRISPR nucleases. Nat. Protoc..

[55] Chen M., Sui T., Yang L. (2022). Live imaging of RNA and RNA splicing in mammalian cells via the dcas13a-SunTag-BiFC system. Biosens. Bioelectron..

[56] Chang C., Ma G., Cheung E. (2022). A programmable system to methylate and demethylate N(6)-methyladenosine (m(6)A) on specific RNA transcripts in mammalian cells. J. Biol. Chem..

[57] Adler B.A., Hessler T., Cress B.F. (2022). Broad-spectrum CRISPR-Cas13a enables efficient phage genome editing. Nat. Microbiol..

[58] Zhou Y., Yang Y., Li X. (2023). Exploiting a conjugative endogenous CRISPR-Cas3 system to tackle multidrug-resistant Klebsiella pneumoniae. EBioMedicine.

[59] Yoshimi K., Takeshita K., Yamayoshi S. (2022). CRISPR-Cas3-based diagnostics for SARS-CoV-2 and influenza virus. iScience.

[60] Hu C., Ni D., Nam K.H. (2022). Allosteric control of type I-A CRISPR-Cas3 complexes and establishment as effective nucleic acid detection and human genome editing tools. Mol. Cell.

[61] Gao Z.L., Herrera-Carrillo E., Berkhout B. (2018). Delineation of the exact transcription termination signal for type 3 polymerase III. Mol. Ther. Nucleic Acids.

[62] Shu-Ichi N., Masao H., Miku K. (2017). Catalytic activities of ribozymes and DNAzymes in water and mixed aqueous media. Catalysts.

[63] Gao Z., Herrera-Carrillo E., Berkhout B. (2018). Improvement of the CRISPR-Cpf1 system with ribozyme-processed crRNA. RNA Biol..

[64] Berkhout B., Gao Z., Herrera-Carrillo E. (2021). Design and evaluation of guide RNA transcripts with a 3'-terminal HDV ribozyme to enhance CRISPR-based gene inactivation. Methods Mol. Biol..

[65] Yamagami R., Kayedkhordeh M., Mathews D.H. (2019). Design of highly active double-pseudoknotted ribozymes: a combined computational and experimental study. Nucleic Acids Res..

[66] Xu L., Zhao L., Gao Y. (2017). Empower multiplex cell and tissue-specific CRISPR-mediated gene manipulation with self-cleaving ribozymes and tRNA. Nucleic Acids Res..

[67] Xie K., Minkenberg B., Yang Y. (2015). Boosting CRISPR/Cas9 multiplex editing capability with the endogenous tRNA-processing system. Proc. Natl. Acad. Sci. U.S.A..

[68] Ding D., Chen K., Chen Y. (2018). Engineering introns to express RNA guides for Cas9- and Cpf1-mediated multiplex genome editing. Mol. Plant.

[69] Dong F., Xie K., Chen Y. (2017). Polycistronic tRNA and CRISPR guide-RNA enables highly efficient multiplexed genome engineering in human cells. Biochem. Biophys. Res. Commun..

[70] Shiraki T., Kawakami K. (2018). A tRNA-based multiplex sgRNA expression system in zebrafish and its application to generation of transgenic albino fish. Sci. Rep..

[71] Yuan Q., Gao X. (2022). Multiplex base- and prime-editing with drive-and-process CRISPR arrays. Nat. Commun..

[72] Wu J., Niu S., Tan M. (2018). Cryo-EM structure of the human ribonuclease P holoenzyme. Cell.

[73] Haurwitz R.E., Jinek M., Wiedenheft B. (2010). Sequence- and structure-specific RNA processing by a CRISPR endonuclease. Science.

[74] Tsai S.Q., Wyvekens N., Khayter C. (2014). Dimeric CRISPR RNA-guided FokI nucleases for highly specific genome editing. Nat. Biotechnol..

[75] Nissim L., Perli S.D., Fridkin A. (2014). Multiplexed and programmable regulation of gene networks with an integrated RNA and CRISPR/Cas toolkit in human cells. Mol. Cell.

[76] Ferreira R., Skrekas C., Nielsen J. (2018). Multiplexed CRISPR/Cas9 genome editing and gene regulation using Csy4 in Saccharomyces cerevisiae. ACS Synth. Biol..

[77] Kurata M., Wolf N.K., Lahr W.S. (2018). Highly multiplexed genome engineering using CRISPR/Cas9 gRNA arrays. PLoS One.

[78] Liu Y., Yang G., Huang S. (2021). Enhancing prime editing by Csy4-mediated processing of pegRNA. Cell Res..

[79] Gao Y., Zhao Y. (2014). Self-processing of ribozyme-flanked RNAs into guide RNAs in vitro and in vivo for CRISPR-mediated genome editing. J. Integr. Plant Biol..

[80] Park H.M., Liu H., Wu J. (2018). Extension of the crRNA enhances Cpf1 gene editing in vitro and in vivo. Nat. Commun..

[81] Bin Moon S., Lee J.M., Kang J.G. (2018). Highly efficient genome editing by CRISPR-Cpf1 using CRISPR RNA with a uridinylate-rich 3'-overhang. Nat. Commun..

[82] Hino T., Omura S.N., Nakagawa R. (2023). An AsCas12f-based compact genome-editing tool derived by deep mutational scanning and structural analysis. Cell.

[83] Li B., Zhao W., Luo X. (2017). Engineering CRISPR-Cpf1 crRNAs and mRNAs to maximize genome editing efficiency. Nat. Biomed. Eng..

[84] Kim H., Lee W.J., Kim C.H. (2022). Highly specific chimeric DNA-RNA-guided genome editing with enhanced CRISPR-Cas12a system. Mol. Ther. Nucleic Acids.

[85] Riesenberg S., Helmbrecht N., Kanis P. (2022). Improved gRNA secondary structures allow editing of target sites resistant to CRISPR-Cas9 cleavage. Nat. Commun..

[86] Gier R.A., Budinich K.A., Evitt N.H. (2020). High-performance CRISPR-Cas12a genome editing for combinatorial genetic screening. Nat. Commun..

[87] Chen R., Cao Y., Liu Y. (2023). Enhancement of a prime editing system via optimal recruitment of the pioneer transcription factor P65. Nat. Commun..

[88] Konermann S., Brigham M.D., Trevino A.E. (2015). Genome-scale transcriptional activation by an engineered CRISPR-Cas9 complex. Nature.

[89] Gao K., Zhang X., Zhang Z. (2022). Transcription-coupled donor DNA expression increases homologous recombination for efficient genome editing. Nucleic Acids Res..

[90] Ha D.I., Lee J.M., Lee N.E. (2020). Highly efficient and safe genome editing by CRISPR-Cas12a using CRISPR RNA with a ribosyl-2 '-O-methylated uridinylate-rich 3 '-overhang in mouse zygotes. Exp. Mol. Med..

[91] Lennox K.A., Behlke M.A. (2020). Chemical modifications in RNA interference and CRISPR/Cas genome editing reagents. Methods Mol. Biol..

[92] Behlke M.A. (2008). Chemical modification of siRNAs for in vivo use. Oligonucleotides.

[93] Mir A., Alterman J.F., Hassler M.R. (2018). Heavily and fully modified RNAs guide efficient SpyCas9-mediated genome editing. Nat. Commun..

[94] Rahdar M., McMahon M.A., Prakash T.P. (2015). Synthetic CRISPR RNA-Cas9-guided genome editing in human cells. Proc. Natl. Acad. Sci. U.S.A..

[95] Mendez-Mancilla A., Wessels H.H., Legut M. (2022). Chemically modified guide RNAs enhance CRISPR-Cas13 knockdown in human cells. Cell Chem. Biol..

[96] Hendel A., Bak R.O., Clark J.T. (2015). Chemically modified guide RNAs enhance CRISPR-Cas genome editing in human primary cells. Nat. Biotechnol..

[97] Suzuki K., Tsunekawa Y., Hernandez-Benitez R. (2016). In vivo genome editing via CRISPR/Cas9 mediated homology-independent targeted integration. Nature.

[98] Liu P., Luk K., Shin M. (2019). Enhanced Cas12a editing in mammalian cells and zebrafish. Nucleic Acids Res..

[99] Luk K., Liu P., Zeng J. (2022). Optimization of nuclear localization signal composition improves CRISPR-Cas12a editing rates in human primary cells. GEN Biotechnol.

[100] Maggio I., Zittersteijn H.A., Wang Q. (2020). Integrating gene delivery and gene-editing technologies by adenoviral vector transfer of optimized CRISPR-Cas9 components. Gene Ther..

[101] Chen Y., Hu Y., Wang X. (2022). Synergistic engineering of CRISPR-Cas nucleases enables robust mammalian genome editing. Innovation.

[102] Duan Z., Liang Y., Sun J. (2024). An engineered Cas12i nuclease that is an efficient genome editing tool in animals and plants. Innovation.

[103] Ma E., Chen K., Shi H. (2022). Improved genome editing by an engineered CRISPR-Cas12a. Nucleic Acids Res..

[104] Guo L.Y., Bian J., Davis A.E. (2022). Multiplexed genome regulation in vivo with hyper-efficient Cas12a. Nat. Cell Biol..

[105] Huang H., Huang G., Tan Z. (2022). Engineered Cas12a-Plus nuclease enables gene editing with enhanced activity and specificity. BMC Biol..

[106] Kleinstiver B.P., Sousa A.A., Walton R.T. (2019). Engineered CRISPR–Cas12a variants with increased activities and improved targeting ranges for gene, epigenetic and base editing. Nat. Biotechnol..

[107] Qi T., Wang Y., Yang Y. (2024). Phage-assisted evolution of compact Cas9 variants targeting a simple NNG PAM. Nat. Chem. Biol..

[108] Schmidheini L., Mathis N., Marquart K.F. (2024). Continuous directed evolution of a compact CjCas9 variant with broad PAM compatibility. Nat. Chem. Biol..

[109] Yang J., Song Y., Deng X. (2023). Engineered LwaCas13a with enhanced collateral activity for nucleic acid detection. Nat. Chem. Biol..

[110] Boccaletto P., Stefaniak F., Ray A. (2022). MODOMICS: a database of RNA modification pathways. 2021 update. Nucleic Acids Res..

[111] Nachtergaele S., He C. (2018). Chemical modifications in the life of an mRNA transcript. Annu. Rev. Genet..

[112] Boo S.H., Kim Y.K. (2020). The emerging role of RNA modifications in the regulation of mRNA stability. Exp. Mol. Med..

[113] Kormann M.S., Hasenpusch G., Aneja M.K. (2011). Expression of therapeutic proteins after delivery of chemically modified mRNA in mice. Nat. Biotechnol..

[114] Vaidyanathan S., Azizian K.T., Haque A. (2018). Uridine depletion and chemical modification increase Cas9 mRNA activity and reduce immunogenicity without HPLC purification. Mol. Ther. Nucleic Acids.

[115] Lieber M.R. (2010). The mechanism of double-strand DNA break repair by the nonhomologous DNA end-joining pathway. Annu. Rev. Biochem..

[116] Budman J., Chu G. (2005). Processing of DNA for nonhomologous end-joining by cell-free extract. EMBO J..

[117] Gu J., Lu H., Tippin B.L. (2007). XRCC4:DNA ligase IV can ligate incompatible DNA ends and can ligate across gaps. EMBO J..

[118] Cermak T., Curtin S.J., Gil-Humanes J. (2017). A multipurpose toolkit to enable advanced genome engineering in plants. Plant Cell.

[119] Yin J., Lu R., Xin C. (2022). Cas9 exo-endonuclease eliminates chromosomal translocations during genome editing. Nat. Commun..

[120] Wu Y., Yuan Q., Zhu Y. (2020). Improving FnCas12a genome editing by exonuclease fusion. CRISPR J..

[121] Lainscek D., Forstneric V., Mikolic V. (2022). Coiled-coil heterodimer-based recruitment of an exonuclease to CRISPR/Cas for enhanced gene editing. Nat. Commun..

[122] Jeacock L., Faria J., Horn D. (2018). Codon usage bias controls mRNA and protein abundance in trypanosomatids. Elife.

[123] Parvathy S.T., Udayasuriyan V., Bhadana V. (2022). Codon usage bias. Mol. Biol. Rep..

[124] Qian W., Zhang J. (2021). Codon usage bias and nuclear mRNA concentration: correlation vs. causation. Proc. Natl. Acad. Sci. U.S.A..

[125] Goulet D.R., Yan Y., Agrawal P. (2023). Codon optimization using a recurrent neural network. J. Comput. Biol..

[126] Karasan O., Sen A., Tiryaki B. (2022). A unifying network modeling approach for codon optimization. Bioinformatics.

[127] Sen A., Kargar K., Akgun E. (2020). Codon optimization: a mathematical programing approach. Bioinformatics.

[128] Koblan L.W., Doman J.L., Wilson C. (2018). Improving cytidine and adenine base editors by expression optimization and ancestral reconstruction. Nat. Biotechnol..

[129] Chau C.H., Steeg P.S., Figg W.D. (2019). Antibody-drug conjugates for cancer. Lancet.

[130] Tsuchikama K., An Z. (2018). Antibody-drug conjugates: recent advances in conjugation and linker chemistries. Protein Cell.

[131] Black S., Phillips D., Hickey J.W. (2021). CODEX multiplexed tissue imaging with DNA-conjugated antibodies. Nat. Protoc..

[132] Wright A.V., Sternberg S.H., Taylor D.W. (2015). Rational design of a split-Cas9 enzyme complex. Proc. Natl. Acad. Sci. U.S.A..

[133] Ling X., Chang L., Chen H. (2021). Improving the efficiency of CRISPR-Cas12a-based genome editing with site-specific covalent Cas12a-crRNA conjugates. Mol. Cell.

[134] Lim D., Sreekanth V., Cox K.J. (2020). Engineering designer beta cells with a CRISPR-Cas9 conjugation platform. Nat. Commun..

[135] Komor A.C., Kim Y.B., Packer M.S. (2016). Programmable editing of a target base in genomic DNA without double-stranded DNA cleavage. Nature.

[136] Komor A.C., Zhao K.T., Packer M.S. (2017). Improved base excision repair inhibition and bacteriophage Mu Gam protein yields C:G-to-T:A base editors with higher efficiency and product purity. Sci. Adv..

[137] Yang C., Dong X., Ma Z. (2022). Pioneer factor improves CRISPR-based C-to-G and C-to-T base editing. Adv. Sci..

[138] Zhang Y., Yun K., Huang H. (2021). Antisense RNA interference-enhanced CRISPR/Cas9 base editing method for improving base editing efficiency in Streptomyces lividans 66. ACS Synth. Biol..

[139] Jiang L., Long J., Yang Y. (2022). Internally inlaid SaCas9 base editors enable window specific base editing. Theranostics.

[140] Zhang G., Song Z., Huang S. (2024). nCas9 engineering for improved target interaction presents an effective strategy to enhance base editing. Adv. Sci..

[141] Nishida K., Arazoe T., Yachie N. (2016). Targeted nucleotide editing using hybrid prokaryotic and vertebrate adaptive immune systems. Science.

[142] Wang X., Li J., Wang Y. (2018). Efficient base editing in methylated regions with a human APOBEC3A-Cas9 fusion. Nat. Biotechnol..

[143] Rees H.A., Komor A.C., Yeh W.-H. (2017). Improving the DNA specificity and applicability of base editing through protein engineering and protein delivery. Nat. Commun..

[144] Wang L., Xue W., Zhang H. (2021). Eliminating base-editor-induced genome-wide and transcriptome-wide off-target mutations. Nat. Cell Biol..

[145] Neugebauer M.E., Hsu A., Arbab M. (2023). Evolution of an adenine base editor into a small, efficient cytosine base editor with low off-target activity. Nat. Biotechnol..

[146] Walton R.T., Christie K.A., Whittaker M.N. (2020). Unconstrained genome targeting with near-PAMless engineered CRISPR-Cas9 variants. Science.

[147] Kweon J., Jang A.-H., Kwon E. (2023). Targeted dual base editing with Campylobacter jejuni Cas9 by single AAV-mediated delivery. Exp. Mol. Med..

[148] Trasanidou D., Barendse P., Bouzetos E. (2023). Efficient genome and base editing in human cells using ThermoCas9. CRISPR J..

[149] Liu Z., Chen S., Jia Y. (2021). Efficient and high-fidelity base editor with expanded PAM compatibility for cytidine dinucleotide. Sci. China Life Sci..

[150] Cheng H., Hao M., Ding B. (2021). Base editing with high efficiency in allotetraploid oilseed rape by A3A-PBE system. Plant Biotechnol. J..

[151] Cho S.-I., Lim K., Hong S. (2024). Engineering TALE-linked deaminases to facilitate precision adenine base editing in mitochondrial DNA. Cell.

[152] Coelho M.A., Cooper S., Strauss M.E. (2023). Base editing screens map mutations affecting interferon-γ signaling in cancer. Cancer Cell.

[153] Kuscu C., Parlak M., Tufan T. (2017). CRISPR-STOP: gene silencing through base-editing-induced nonsense mutations. Nat. Methods.

[154] Han W., Qiu H.Y., Sun S. (2023). Base editing of the HBG promoter induces potent fetal hemoglobin expression with no detectable off-target mutations in human HSCs. Cell Stem Cell.

[155] Katti A., Vega-Pérez A., Foronda M. (2024). Generation of precision preclinical cancer models using regulated in vivo base editing. Nat. Biotechnol..

[156] Geurts M.H., Gandhi S., Boretto M.G. (2023). One-step generation of tumor models by base editor multiplexing in adult stem cell-derived organoids. Nat. Commun..

[157] Pan J.S., Lin Z.S., Wen J.C. (2021). Application of the modified cytosine base-editing in the cultured cells of bama minipig. Biotechnol. Lett..

[158] Song R., Wang Y., Zheng Q. (2022). One-step base editing in multiple genes by direct embryo injection for pig trait improvement. Sci. China Life Sci..

[159] Wu Y., He Y., Sretenovic S. (2022). CRISPR-BETS: a base-editing design tool for generating stop codons. Plant Biotechnol. J..

[160] Yao T., Yuan G., Lu H. (2023). CRISPR/Cas9-based gene activation and base editing in Populus. Hortic. Res..

[161] Gaudelli N.M., Komor A.C., Rees H.A. (2017). Programmable base editing of A•T to G•C in genomic DNA without DNA cleavage. Nature.

[162] Richter M.F., Zhao K.T., Eton E. (2020). Phage-assisted evolution of an adenine base editor with improved Cas domain compatibility and activity. Nat. Biotechnol..

[163] Chen L., Zhang S., Xue N. (2023). Engineering a precise adenine base editor with minimal bystander editing. Nat. Chem. Biol..

[164] Tu T., Song Z., Liu X. (2022). A precise and efficient adenine base editor. Mol. Ther..

[165] Li G., Cheng Y., Li Y. (2023). A novel base editor SpRY-ABE8e(F148A) mediates efficient A-to-G base editing with a reduced off-target effect. Mol. Ther. Nucleic Acids.

[166] Zhao D., Gao X., Zhou J. (2023). Engineered domain-inlaid Nme2Cas9 adenine base editors with increased on-target DNA editing and targeting scope. BMC Biol..

[167] Shin H.R., See J.-E., Kweon J. (2021). Small-molecule inhibitors of histone deacetylase improve CRISPR-based adenine base editing. Nucleic Acids Res..

[168] Jeong Y.K., Lee S., Hwang G.-H. (2021). Adenine base editor engineering reduces editing of bystander cytosines. Nat. Biotechnol..

[169] Chen L., Zhang S., Xue N. (2023). Engineering a precise adenine base editor with minimal bystander editing. Nat. Chem. Biol..

[170] Chen S., Liu Z., Xie W. (2022). Compact Cje3Cas9 for efficient in vivo genome editing and adenine base editing. CRISPR J..

[171] Li G., Cheng Y., Li Y. (2023). A novel base editor SpRY-ABE8eF148A mediates efficient A-to-G base editing with a reduced off-target effect. Mol. Ther. Nucleic Acids.

[172] Zhao D., Jiang G., Li J. (2022). Imperfect guide-RNA (igRNA) enables CRISPR single-base editing with ABE and CBE. Nucleic Acids Res..

[173] Choi E., Hwang H.-Y., Kwon E. (2022). Expanded targeting scope of LbCas12a variants allows editing of multiple oncogenic mutations. Mol. Ther. Nucleic Acids.

[174] Tan J., Zeng D., Zhao Y. (2022). PhieABEs: a PAM-less/free high-efficiency adenine base editor toolbox with wide target scope in plants. Plant Biotechnol. J..

[175] Hu Y., Han L., Mo Q. (2024). Engineering miniature CRISPR-Cas Un1Cas12f1 for efficient base editing. Mol. Ther. Nucleic Acids.

[176] Brooks D.L., Whittaker M.N., Said H. (2024). A base editing strategy using mRNA-LNPs for in vivo correction of the most frequent phenylketonuria variant. HGG Adv.

[177] Lebek S., Caravia X.M., Straub L.G. (2024). CRISPR-Cas9 base editing of pathogenic CaMKIIδ improves cardiac function in a humanized mouse model. J. Clin. Invest..

[178] Li G., Liu X., Huang S. (2019). Efficient generation of pathogenic A-to-G mutations in human tripronuclear embryos via ABE-mediated base editing. Mol. Ther. Nucleic Acids.

[179] Ryu S.M., Koo T., Kim K. (2018). Adenine base editing in mouse embryos and an adult mouse model of Duchenne muscular dystrophy. Nat. Biotechnol..

[180] Hu L., Zhai Y., Xu L. (2022). Precise A∙T to G∙C base editing in the allotetraploid rapeseed (Brassica napus L.) genome. J. Cell. Physiol..

[181] Zhou S., Ding Y., Liu J. (2020). Highly efficient generation of sheep with a defined FecB(B) mutation via adenine base editing. Genet. Sel. Evol..

[182] Zhao D., Li J., Li S. (2021). Glycosylase base editors enable C-to-A and C-to-G base changes. Nat. Biotechnol..

[183] Kurt I.C., Zhou R., Iyer S. (2021). CRISPR C-to-G base editors for inducing targeted DNA transversions in human cells. Nat. Biotechnol..

[184] Chen L., Zhu B., Ru G. (2023). Re-engineering the adenine deaminase TadA-8e for efficient and specific CRISPR-based cytosine base editing. Nat. Biotechnol..

[185] Tong H., Wang X., Liu Y. (2023). Programmable A-to-Y base editing by fusing an adenine base editor with an N-methylpurine DNA glycosylase. Nat. Biotechnol..

[186] Anzalone A.V., Randolph P.B., Davis J.R. (2019). Search-and-replace genome editing without double-strand breaks or donor DNA. Nature.

[187] Liu Y., Yang G., Huang S. (2021). Enhancing prime editing by Csy4-mediated processing of pegRNA. Cell Res..

[188] Li X., Zhou L., Gao B.-Q. (2022). Highly efficient prime editing by introducing same-sense mutations in pegRNA or stabilizing its structure. Nat. Commun..

[189] Nelson J.W., Randolph P.B., Shen S.P. (2022). Engineered pegRNAs improve prime editing efficiency. Nat. Biotechnol..

[190] Li X., Wang X., Sun W. (2022). Enhancing prime editing efficiency by modified pegRNA with RNA G-quadruplexes. J. Mol. Cell Biol..

[191] Feng Y., Liu S., Mo Q. (2023). Enhancing prime editing efficiency and flexibility with tethered and split pegRNAs. Protein Cell.

[192] Adikusuma F., Lushington C., Arudkumar J. (2021). Optimized nickase- and nuclease-based prime editing in human and mouse cells. Nucleic Acids Res..

[193] Lee J., Lim K., Kim A. (2023). Prime editing with genuine Cas9 nickases minimizes unwanted indels. Nat. Commun..

[194] Doman J.L., Pandey S., Neugebauer M.E. (2023). Phage-assisted evolution and protein engineering yield compact, efficient prime editors. Cell.

[195] Chen P.J., Hussmann J.A., Yan J. (2021). Enhanced prime editing systems by manipulating cellular determinants of editing outcomes. Cell.

[196] Liu P., Liang S.-Q., Zheng C. (2021). Improved prime editors enable pathogenic allele correction and cancer modelling in adult mice. Nat. Commun..

[197] Weber Y., Böck D., Ivașcu A. (2024). Enhancing prime editor activity by directed protein evolution in yeast. Nat. Commun..

[198] Ni P., Zhao Y., Zhou X. (2023). Efficient and versatile multiplex prime editing in hexaploid wheat. Genome Biol..

[199] Liang Z., Wu Y., Guo Y. (2023). Addition of the T5 exonuclease increases the prime editing efficiency in plants. J. Genet. Genomics.

[200] Truong D.-J.J., Geilenkeuser J., Wendel S.V. (2024). Exonuclease-enhanced prime editors. Nat. Methods.

[201] Zhang H., Ma J., Wu Z. (2024). BacPE: a versatile prime-editing platform in bacteria by inhibiting DNA exonucleases. Nat. Commun..

[202] Maeder M.L., Linder S.J., Cascio V.M. (2013). CRISPR RNA-guided activation of endogenous human genes. Nat. Methods.

[203] Chavez A., Scheiman J., Vora S. (2015). Highly efficient Cas9-mediated transcriptional programming. Nat. Methods.

[204] Weltner J., Balboa D., Katayama S. (2018). Human pluripotent reprogramming with CRISPR activators. Nat. Commun..

[205] Vora S., Cheng J., Xiao R. (2018). Rational design of a compact CRISPR-Cas9 activator for AAV-mediated delivery. bioRxiv.

[206] Zhang X., Wang W., Shan L. (2017). Gene activation in human cells using CRISPR/Cpf1-p300 and CRISPR/Cpf1-SunTag systems. Protein Cell.

[207] Tanenbaum Marvin E., Gilbert Luke A., Qi Lei S. (2014). A protein-tagging system for signal amplification in gene expression and fluorescence imaging. Cell.

[208] Qi Lei S., Larson Matthew H., Gilbert Luke A. (2013). Repurposing CRISPR as an RNA-guided platform for sequence-specific control of gene expression. Cell.

[209] Gilbert L.A., Larson M.H., Morsut L. (2013). CRISPR-mediated modular RNA-guided regulation of transcription in eukaryotes. Cell.

[210] Iyengar S., Farnham P.J. (2011). KAP1 protein: an enigmatic master regulator of the genome. J. Biol. Chem..

[211] Yeo N.C., Chavez A., Lance-Byrne A. (2018). An enhanced CRISPR repressor for targeted mammalian gene regulation. Nat. Methods.

[212] Alerasool N., Segal D., Lee H. (2020). An efficient KRAB domain for CRISPRi applications in human cells. Nat. Methods.

[213] Wang L., Wang E., Prado Balcazar J. (2021). Chromatin remodeling of colorectal cancer liver metastasis is mediated by an HGF-PU.1-DPP4 axis. Adv. Sci..

[214] Tarjan D.R., Flavahan W.A., Bernstein B.E. (2019). Epigenome editing strategies for the functional annotation of CTCF insulators. Nat. Commun..

[215] Karvelis T., Bigelyte G., Young J.K. (2020). PAM recognition by miniature CRISPR–Cas12f nucleases triggers programmable double-stranded DNA target cleavage. Nucleic Acids Res..

[216] Takeda S.N., Nakagawa R., Okazaki S. (2021). Structure of the miniature type V-F CRISPR-Cas effector enzyme. Mol. Cell.

[217] Kim D.Y., Lee J.M., Moon S.B. (2022). Efficient CRISPR editing with a hypercompact Cas12f1 and engineered guide RNAs delivered by adeno-associated virus. Nat. Biotechnol..

[218] Kong X., Zhang H., Li G. (2023). Engineered CRISPR-OsCas12f1 and RhCas12f1 with robust activities and expanded target range for genome editing. Nat. Commun..

[219] Wu T., Liu C., Zou S. (2023). An engineered hypercompact CRISPR-Cas12f system with boosted gene-editing activity. Nat. Chem. Biol..

[220] Kato K., Okazaki S., Kannan S. (2022). Structure of the IscB–ωRNA ribonucleoprotein complex, the likely ancestor of CRISPR-Cas9. Nat. Commun..

[221] Han D., Xiao Q., Wang Y. (2023). Development of miniature base editors using engineered IscB nickase. Nat. Methods.

[222] Sasnauskas G., Tamulaitiene G., Druteika G. (2023). TnpB structure reveals minimal functional core of Cas12 nuclease family. Nature.

[223] Karvelis T., Druteika G., Bigelyte G. (2021). Transposon-associated TnpB is a programmable RNA-guided DNA endonuclease. Nature.

[224] Wang M., Sun Z., Liu Y. (2024). Hypercompact TnpB and truncated TnpB systems enable efficient genome editing in vitro and in vivo. Cell Discov.

[225] Li Z., Guo R., Sun X. (2024). Engineering a transposon-associated TnpB-ωRNA system for efficient gene editing and phenotypic correction of a tyrosinaemia mouse model. Nat. Commun..

[226] Ren K., Zhou F., Zhang F. (2024). Discovery and structural mechanism of DNA endonucleases guided by RAGATH-18-derived RNAs. Cell Res..

[227] Jiang K., Lim J., Sgrizzi S. (2023). Programmable RNA-guided DNA endonucleases are widespread in eukaryotes and their viruses. Sci. Adv..

[228] Yoon P.H., Skopintsev P., Shi H. (2023). Eukaryotic RNA-guided endonucleases evolved from a unique clade of bacterial enzymes. Nucleic Acids Res..

[229] Saito M., Xu P., Faure G. (2023). Fanzor is a eukaryotic programmable RNA-guided endonuclease. Nature.

[230] Strecker J., Ladha A., Gardner Z. (2019). RNA-guided DNA insertion with CRISPR-associated transposases. Science.

[231] Cui Y., Dong H., Tong B. (2022). A versatile Cas12k-based genetic engineering toolkit (C12KGET) for metabolic engineering in genetic manipulation-deprived strains. Nucleic Acids Res..

[232] Cheng Z.H., Wu J., Liu J.Q. (2022). Repurposing CRISPR RNA-guided integrases system for one-step, efficient genomic integration of ultra-long DNA sequences. Nucleic Acids Res..

[233] Tou C.J., Orr B., Kleinstiver B.P. (2023). Precise cut-and-paste DNA insertion using engineered type V-K CRISPR-associated transposases. Nat. Biotechnol..

[234] Cong L., Ran F.A., Cox D. (2013). Multiplex genome engineering using CRISPR/Cas systems. Science.

[235] Zhang X.H., Tee L.Y., Wang X.G. (2015). Off-target effects in CRISPR/Cas9-mediated genome engineering. Mol. Ther. Nucleic Acids.

[236] Doench J.G., Fusi N., Sullender M. (2016). Optimized sgRNA design to maximize activity and minimize off-target effects of CRISPR-Cas9. Nat. Biotechnol..

[237] Fu Y., Sander J.D., Reyon D. (2014). Improving CRISPR-Cas nuclease specificity using truncated guide RNAs. Nat. Biotechnol..

[238] Zhang J.-P., Li X.-L., Neises A. (2016). Different effects of sgRNA length on CRISPR-mediated gene knockout efficiency. Sci. Rep..

[239] Pattanayak V., Lin S., Guilinger J.P. (2013). High-throughput profiling of off-target DNA cleavage reveals RNA-programmed Cas9 nuclease specificity. Nat. Biotechnol..

[240] Ran F.A., Hsu P.D., Lin C.Y. (2013). Double nicking by RNA-guided CRISPR Cas9 for enhanced genome editing specificity. Cell.

[241] Slaymaker I.M., Gao L., Zetsche B. (2016). Rationally engineered Cas9 nucleases with improved specificity. Science.

[242] Kleinstiver B.P., Pattanayak V., Prew M.S. (2016). High-fidelity CRISPR–Cas9 nucleases with no detectable genome-wide off-target effects. Nature.

[243] Casini A., Olivieri M., Petris G. (2018). A highly specific SpCas9 variant is identified by in vivo screening in yeast. Nat. Biotechnol..

[244] Tan Y., Chu A.H.Y., Bao S. (2019). Rationally engineered Staphylococcus aureus Cas9 nucleases with high genome-wide specificity. Proc. Natl. Acad. Sci. U.S.A..

[245] Xie H., Ge X., Yang F. (2020). High-fidelity SaCas9 identified by directional screening in human cells. PLoS Biol..

[246] Lv J., Xi H., Lv X. (2022). Two high-fidelity variants: efSaCas9 and SaCas9-HF, which one is better?. Gene Ther..

